# Mild and repetitive mild traumatic brain injury: Changes in microglial cells and synapses

**DOI:** 10.4103/NRR.NRR-D-25-00902

**Published:** 2025-12-30

**Authors:** Adna Smajkan, Elizabeth Naranjo-Cinto, Florence M. Bareyre

**Affiliations:** 1 Institute of Clinical Neuroimmunology, University Hospital, LMU Munich, Munich, Germany; 2 Biomedical Center Munich (BMC), Medical Faculty, LMU Munich, Planegg-Martinsried, Germany; 3 Graduate School of Systemic Neurosciences, Ludwig-Maximilians-Universitaet Muenchen, Planegg-Martinsried, Germany; 4 Elite MSc. Biomedical Neuroscience, Technische Universitaet Muenchen, Munich, Germany; 5 Munich Cluster of Systems Neurology (SyNergy), Munich, Germany

**Keywords:** animal models, biomarkers, concussion, connectivity, microglia cells, microglia-mediated synapse engulfment, repetitive concussion, synapse loss

## Abstract

Mild traumatic brain injury results from external mechanical forces to the head. Repetitive mild traumatic brain injury, characterized by multiple concussive events over time, is increasingly recognized in contact sports or domestic abuse. Repetitive injuries are associated with a greater risk of cumulative deficits and chronic neurodegenerative conditions. After a period of large focus on gross morphological changes following mild traumatic brain injury and repetitive mild traumatic brain injury, recent research is exploring more subtle yet critical changes at the synaptic and microglial levels. Novel findings indicate that even a single mild traumatic brain injury can induce transient alterations in synaptic function, including increased excitatory neurotransmission and disrupted synaptic plasticity. Parallel to synaptic changes, microglial cells, the brain’s resident immune cells, undergo rapid and prolonged activation after mild traumatic brain injury, including morphological transformation and functional activation. In repetitive mild traumatic brain injury, microglial priming is more pronounced, leading to sustained neuroimmune dysregulation and heightened and persistent vulnerability. In this review, we will summarize the current literature on mild traumatic brain injury and repetitive mild traumatic brain injury with a specific emphasis on microglial and synaptic changes.

## Introduction

Traumatic brain injury (TBI) is a major public health concern and one of the leading causes of death and long-term disability worldwide. Each year, an estimated 69 million people experience TBI globally, with mild cases accounting for the majority of injuries (Dewan et al., 2018). The burden of mild TBI (mTBI) is particularly high among vulnerable populations, including children, adolescents, older adults, and victims of abuse. In addition to its clinical consequences, mTBI poses a substantial economic and social burden due to long-term disability, loss of productivity, and strain on healthcare systems (Berwick et al., 2022; Al Fudhaili et al., 2025).

TBI results from external mechanical forces, such as a direct blow to the head or other acceleration/deceleration forces, with or without loss of consciousness. Alterations in brain function can manifest through various mild clinical signs, such as loss or reduction of consciousness, headaches, dizziness, fatigue, sleep disruption, and cognitive difficulties. In addition, individuals may experience behavioral symptoms such as guilt, irritability, worry, mental overload, and a sense of emptiness. Although these symptoms often improve within days to weeks, they are not always recognized right away, leading to many mild traumatic brain injuries going undiagnosed, especially when symptoms are mild or appear later (Jenkins et al., 2023; Vutakuri, 2023). mTBI is typically diagnosed based on the immediate onset of clinical symptoms, with recent advances in neuroimaging and emerging blood-based biomarkers offering promising non-invasive tools to improve diagnostic accuracy and facilitate the identification of subtle or delayed injury-related changes (Wilde et al., 2022). While most patients recover fully from mTBI, the risk of complications increases with repeated injuries. Repetitive mild TBI (rmTBI) particularly impacts athletes and victims of abuse. The accumulation of mild TBI is associated with an increased risk of long-term neurodegenerative diseases such as chronic traumatic encephalopathy (CTE), Alzheimer-like dementia, and Parkinsonism (DeKosky et al., 2013; McKee et al., 2016; Katsumoto et al., 2019; Mackay et al., 2019). rmTBI also increases the severity and duration of acute symptoms, thereby complicating recovery and contributing to long-lasting impairments. Furthermore, individual characteristics such as age, sex, genetic predispositions, and coexisting medical conditions can significantly alter both the acute response and long-term outcomes (Xiong et al., 2013; Bennett et al., 2016). This variability underscores the importance of prevention and personalized approaches in the management and rehabilitation of single and repetitive mild TBI, aiming to address not only physical and cognitive impairments but also emotional and psychosocial challenges that may emerge over time (Prince and Bruhns, 2017; van der Horn et al., 2020).

## Search Strategy

**Figure 1:**
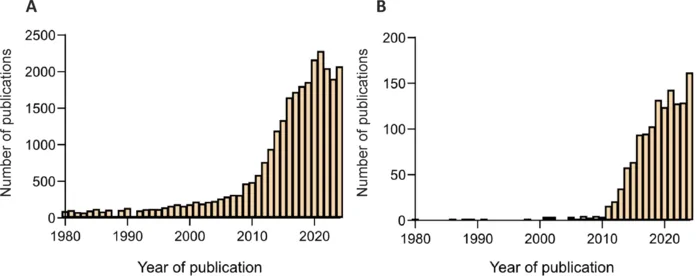
Histograms generated from PubMed. This histogram depicts the number of publications per year from 1980 to 2024 for the query “concussion or mild traumatic brain injury” (A) and “repetitive concussion or repetitive mild traumatic brain injury” (B). Created with BioRender.com.

We performed a comprehensive literature search in the PubMed database, covering articles published from 2010 to 2025, while also including key earlier studies (dating back to 1928) to provide a historical perspective on the evolving understanding of mild and repetitive mild traumatic brain injury. The search included the following Medical Subject Headings (MeSH) terms: “Mild Traumatic Brain Injury” [Mesh] OR “Repetitive Mild Traumatic Brain Injury” [Mesh] OR “Concussion” [Mesh] OR “Repetitive Concussion” [Mesh] AND (“Microglia” [Mesh] OR “Glial Activation” [Mesh] OR “Synaptic Alterations” OR “Neuronal Dysfunction” [Mesh] OR “Connectivity Alterations” OR “Animal Models”). We screened titles and abstracts for studies addressing cellular and circuit-level changes induced by single or repetitive mild TBI, with a particular focus on synaptic remodeling and network dynamics, and glial and neuronal mechanisms following single or repetitive mild TBI. Our search highlights the growing interest in mild/repetitive mild TBI in the last few years and the strong development of research in this field (**Figure 1**).

## Mild Traumatic Brain Injury: Global Burden, Classification, and Underlying Causes

TBI classification has evolved significantly over the past five decades. The foundation for modern classification was laid with the development of the Glasgow Coma Scale (GCS) in 1974 by Teasdale and Jennett. The GCS provided a standardized method for assessing consciousness in patients with acute brain injury, using a scoring system based on eye, verbal, and motor responses (Teasdale and Jennett, 1974). This scale quickly became a cornerstone of clinical assessment and research. In the 1980s and early 1990s, researchers began using GCS ranges alongside clinical indicators such as loss of consciousness and post-traumatic amnesia to categorize TBI as mild, moderate, or severe (Jennett and Teasdale, 1981; Rimel et al., 1981).

### Diagnostic criteria for mild traumatic brain injury

The classification of mTBI was formalized in the early 1990s, when the American Congress of Rehabilitation Medicine (ACRM) proposed one of the first widely accepted diagnostic criteria. In 1993, the ACRM defined mTBI as involving one or more of the following: loss of consciousness lasting up to 30 minutes, post-traumatic amnesia lasting less than 24 hours, an altered mental state (e.g., confusion or disorientation), or transient neurological deficits, within the context of a GCS score of 13 to 15 (Mild Traumatic Brain Injury Committee of the ACRM, 1993).

This framework contributed significantly to the recognition and standardization of mild TBI diagnosis, and it was subsequently adopted by major health organizations, including the Centers for Disease Control and Prevention and the World Health Organization.

However, the traditional framework has since faced criticism for its limited sensitivity to the heterogeneity and long-term consequences of mTBI, particularly in cases where structural imaging appears normal but cognitive or emotional symptoms persist (Iverson and Lange, 2003; Belanger and Vanderploeg, 2005; Yue et al., 2013; McInnes et al., 2017). Findings from the Transforming Research and Clinical Knowledge in TBI study (TRACK-TBI) further support these concerns, revealing that nearly 30% of patients classified as having mild TBI based on standard clinical criteria exhibited abnormalities on advanced magnetic resonance imaging (MRI) scans (Yue et al., 2013). These discrepancies suggest that reliance on GCS, loss of consciousness, and post-traumatic amnesia alone may underestimate injury severity and fail to identify individuals at risk for persistent or progressive dysfunction.

### “Silent epidemic” and global burden

Despite its classification as “mild,” the high prevalence of these injuries, which account for 70% to 90% of TBI cases globally, makes them a major public health concern. The most recent publicly available global estimates of the burden of mild TBI were derived from the Global Burden of Disease Study (GBD), covering the years 1990 to 2019 (GBD 2019 Disease and Injury Incidence and Prevalence Collaborators, 2020).

Wu et al. (2024) conducted a cross-sectional analysis focused on mild TBI, using data extracted from these studies. According to their findings, in 2019, mTBI accounted for approximately 12.27 million new cases globally, with 11.48 million prevalent cases and about 1.37 million years lived with disability. Sex- and age-specific analyses revealed that the prevalence of mTBI was higher among males and older adults. Additionally, regions with a low sociodemographic index experienced a higher burden.

Although the 2019 estimates remain the most detailed for mTBI specifically, the authors also reported that overall TBI cases increased to approximately 20.84 million by 2021. Despite this rise in absolute numbers, age-standardized rates of incidence, prevalence, and years lived with disability have declined globally, suggesting improvements in prevention and clinical management (Maas et al., 2022). Because the symptoms of mild TBI are typically subtle or delayed, making them difficult to recognize, diagnose, and treat effectively, mild TBI is often referred to as the “silent epidemic,” and patients may not seek help.

### Etiology of mild traumatic brain injury

mTBI can result from a wide range of external forces and often occurs during routine daily activities. The most common cause is falls, particularly among older adults and young children. Other frequent causes include road traffic accidents involving motor vehicles, motorcyclists, cyclists, and pedestrians. mTBI can also result from physical violence, transport-related incidents, impacts from mechanical forces, and other unintentional injuries. These diverse mechanisms reflect the fact that mTBI can affect individuals in almost any setting, from the home and workplace to streets and public spaces (Menon et al., 2010; Maas et al., 2017; Centers for Disease Control and Prevention, 2021).

While many cases of mTBI occur as single, non-repeating events, certain populations face repeated exposure to mild head trauma, which may lead to repetitive mTBI (rmTBI). This is particularly common among individuals engaged in contact sports, military personnel, and those experiencing chronic domestic violence. Recurrent injuries, even if each event is considered mild, can have cumulative effects on brain health, increasing the risk of prolonged symptoms, cognitive impairment, and neurodegenerative conditions such as CTE (McKee et al., 2013). Recognition of rmTBI is critical, as it shifts the focus from short-term recovery to the long-term consequences of repeated brain trauma.

### Specificities of repetitive mild brain injuries

**Figure 2:**
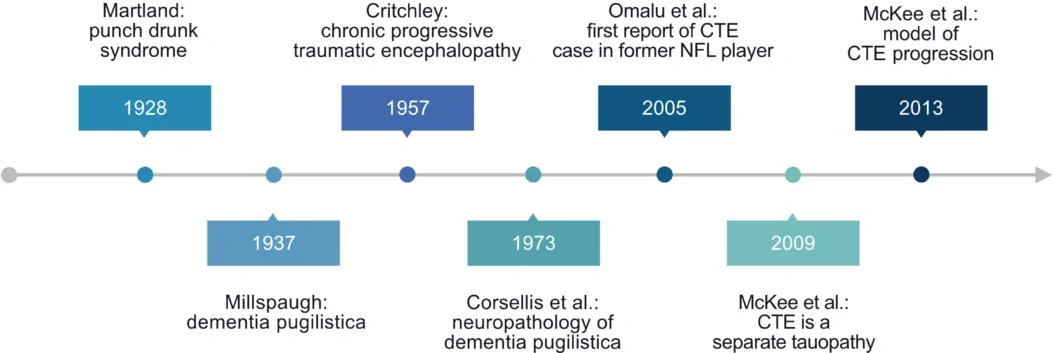
Timeline of investigations of the chronic consequences of repetitive mild traumatic brain injury (rmTBI) over the years, with an emphasis on the behavioral and pathological hallmarks of rmTBI. Created with BioRender.com. CTE: Chronic traumatic encephalopathy; NFL: National Football League.

While a single mTBI is typically considered transient, growing evidence shows that repeated concussions can lead to more serious and lasting neurological consequences. For almost a century, it has been known that repeated head trauma can result in progressive neurological decline. The first medical recognition of the long-term consequences of repeated concussions came in 1928 when Dr. Harrison Martland described “punch drunk syndrome,” a condition observed in professional boxers who exhibited motor and cognitive disturbances. His work laid the foundation for the current understanding of CTE, a condition increasingly identified in athletes, veterans, and others exposed to repeated mild brain injuries (Martland, 1928; **Figure 2**).

Nearly a decade after Martland’s initial observations, Millspaugh (1937) proposed the term “*dementia pugilistica*” to better characterize the long-term neurological deterioration seen in boxers exposed to repeated head trauma. While Martland’s concept of “punch-drunk syndrome” focused on short-term and inconsistent symptoms, such as clumsiness, slurred speech, and emotional instability (Martland, 1928), Millspaugh reframed the condition as a chronic and progressive brain disorder, suggesting permanent neurological damage rather than temporary effects from recent trauma (**Figure 2**).

Building on these early findings, research in the 1930s and 1940s further supported the link between repeated head trauma and lasting brain damage. In 1934, H.L. Parker provided some of the first pathological evidence to support this idea. Through post-mortem examinations of boxers’ brains, Parker identified clear signs of structural injury, including small hemorrhages and multiple localized lesions (Parker, 1934). This work established a direct connection between repeated head trauma and specific brain pathologies. Around the same time, Jokl and Guttmann (1933) conducted neurological and psychiatric assessments of boxers, observing cognitive and behavioral impairments consistent with a trauma-induced dementia (Castellani et al., 2017).

From the mid-20^th^ century through the 1980s, the long-term consequences of repetitive head trauma received little attention, largely due to limited diagnostic tools and a general reluctance to critically examine the risks associated with popular contact sports. While dementia pugilistica remained a recognized condition in boxing circles, broader medical engagement was rare, and only a few key studies provided early clinical and pathological insights (Critchley, 1957; Corsellis et al., 1973; **Figure 2**)

It was not until the early 2000s that scientific and public awareness began to shift meaningfully. This turning point came in 2005, when Bennet Omalu published the first modern case of CTE in former National Football League player Mike Webster, renewing attention to the cumulative impact of repetitive head trauma (Omalu et al., 2005). This study reopened the scientific and public debate around the dangers of repeated head trauma, expanded the scope beyond boxing, and laid the foundation for modern understanding and policy regarding CTE in athletes.

The next major step came in 2009, when McKee et al. published a seminal study defining CTE as a distinct tauopathy, characterized by a unique pattern of tau deposition that set it apart from other neurodegenerative diseases such as Alzheimer’s disease (McKee et al., 2009). Subsequent work by McKee et al. (2013) introduced a four-stage model of disease progression, correlating increasing tau burden with worsening clinical symptoms, from mood and behavioral changes to full-blown dementia. Since then, many studies have focused on rmTBI, the functional and structural brain changes that follow, and the diagnostic tools to study them (**Figure 2**).

Despite advancements in describing CTE pathology, there remained a lack of standardization necessary for clinical and research consensus. A major step toward developing formal diagnostic criteria occurred in 2016 during the first National Institute of Neurological Disorders and Stroke and the National Institute of Biomedical Imaging and Bioengineering consensus meeting, where several experienced neuropathologists gathered to evaluate and agree upon a specific diagnosis for CTE. The clear conclusion was that the pathology of CTE is distinct from that of other neurodegenerative diseases, including Alzheimer’s disease, progressive supranuclear palsy, and corticobasal degeneration, as well as less common conditions such as Guam Parkinsonism-Dementia Complex, argyrophilic grain disease, and primary age-related tauopathy. The panel described the distinctive lesion of CTE as the atypical accumulation of abnormal tau protein in both neurons and astrocytes, particularly clustered around small blood vessels, in an irregular pattern in the depths of cortical sulci (McKee et al., 2016).

Improvements in the diagnostic precision of CTE were established during the second National Institute of Neurological Disorders and Stroke and National Institute of Biomedical Imaging and Bioengineering consensus meeting in 2021. While the original definition was reaffirmed, the panel introduced important refinements to enhance specificity. Notably, they emphasized that neuronal tau must be present in the lesion, with astrocytic tau alone, commonly observed in aging-related tau astrogliopathy, being insufficient. Additionally, the lesion must be in the deeper cortical layers, rather than limited to superficial or subpial regions. These updates were especially important for reducing diagnostic uncertainty in older individuals, where age-related tau pathologies such as aging-related tau astrogliopathy and primary age-related tauopathy can closely mimic early features of CTE (Bieniek et al., 2021).

## Animal Models of Mild Traumatic Brain Injury

Animal models play a crucial role in advancing our understanding of mild traumatic brain injury, with various models using different approaches to induce injury. Large animal models provide several advantages, including brain anatomy, biomechanics, and white matter composition that more closely reflect human physiology (Cullen et al., 2016; Vink, 2018). However, rodent models are more widely used due to their low cost, genetic tractability, and suitability for large-scale, well-controlled experiments (Xiong et al., 2013; Shultz et al., 2017).

### Fluid percussion model

The origins of the fluid percussion injury (FPI) model can be traced to the work of Lindgren and Rinder (1966), who first described the use of controlled pressure pulses delivered through a fluid-filled system to study brain injury in a rabbit model. This approach was initially termed “percussion concussion.” However, the more standardized FPI model, adapted for rats and widely used in current mTBI studies, originates from Dixon et al. (1987). In their work, they developed a piston-driven system that delivered a brief, well-controlled fluid pulse directly to the exposed dura after a craniotomy, allowing for precise control of injury severity and greatly improving reproducibility.

Different types of injuries can be produced depending on the location of pulse delivery: when applied centrally over the sagittal suture between bregma and lambda (midline FPI), the model typically produces diffuse brain injury; when applied laterally over the parietal cortex (lateral FPI), it produces a mixed focal and diffuse injury. Lateral FPI is the more widely used variant, particularly in studies of mTBI/rmTBI, due to its ability to model both focal and diffuse pathological features (Thompson et al., 2005). The resulting injury typically leads to transient blood–brain barrier (BBB) disruption, neuroinflammation, and subtle cognitive and motor deficits, without visible structural damage, closely modeling key features of human concussion (Eakin et al., 2015).

The fluid percussion injury induces displacement and deformation of brain tissue, with injury severity readily modulated by adjusting the pendulum height, which controls the force of the resulting pressure pulse (Xiong et al., 2013). Additionally, craniotomy positioning is critical in this model, as even small variations can significantly affect the size, location, and type of brain injury, making precise placement essential for reproducibility and outcome interpretation (Vink et al., 2001).

This model offers several advantages, including the ability to easily adjust injury severity by varying the force of the fluid pulse. It produces both diffuse axonal injury and focal tissue damage, and can reliably induce behavioral deficits. However, FPI does not replicate the mechanical impact forces of clinical TBI, and its injury dynamics are influenced by species-specific brain geometry, making the model less suited for detailed biomechanical analysis (Xiong et al., 2013).

### Weight drop model

The weight-drop model is a simple and widely used experimental TBI model in rodents that employs the gravitational force of a free-falling guided weight to produce brain injury (Morales et al., 2005). The development of weight-drop models for rodents was inspired in part by early impact acceleration studies in primates (Gennarelli et al., 1982), which demonstrated that diffuse axonal injury could result from rapid angular head acceleration. Building on this concept, Marmarou et al. (1994) developed what became the most widely adopted rodent weight-drop model of diffuse brain injury. In this model, a free-falling weight is dropped onto a stainless-steel disk affixed to the intact skull, inducing rapid head acceleration and widespread diffuse axonal injury. Injury severity is adjustable by varying the weight and drop height. Although it was initially used to study moderate to severe TBI, it was later adapted to better replicate the subtle pathology of mTBI.

To better model clinically relevant closed head injury, Shapira et al. (1988) adapted the weight-drop approach by immobilizing the head of the rat on a rigid platform during impact, thereby minimizing inertial brain motion and enabling a controlled and reproducible closed head injury model. This approach was subsequently refined to yield a mTBI model characterized by focal blunt injury, BBB disruption, glial activation, and persistent neurobehavioral deficits.

Weight-drop models are simple, low-cost, and allow graded injury, making them usable for mTBI and rmTBI. However, variability in impact velocity and the potential risk of weight rebound can lead to inconsistent injury outcomes (Xiong et al., 2013). Further advancements included the development of the Maryland model, which introduced frontal impacts that induce anterior–posterior and rotational head acceleration, providing a more clinically relevant approximation of real-world closed head injury mechanics (Kilbourne et al., 2009). Recent studies continue to employ weight-drop models to explore region-specific molecular and transcriptomic responses to TBI (Chakraborty et al., 2021; Zhuang et al., 2025), also in freely moving animals (Kane et al., 2012).

### Controlled cortical impact

The controlled cortical impact (CCI) model was adapted from methods originally used in experimental spinal cord injury studies (Anderson, 1982) and later modified to produce controlled, reproducible cortical deformation in experimental TBI models. It was first introduced for brain injury in ferrets by Lighthall (1988) and subsequently adapted for use in rodents (Dixon et al., 1991). The model employs either a pneumatic or electromagnetic device to accelerate a rigid impactor, which is directed to strike the exposed dura mater, generating controlled deformation of the underlying cortex. The injury is highly reproducible, scalable, and applicable across multiple species (Lighthall, 1988; Lighthall et al., 1990; Dixon et al., 1991; Smith et al., 1995; Manley et al., 2006; King et al., 2010).

A key advantage of the CCI model is its precise control over mechanical parameters such as impact depth, velocity, and dwell time, which can be finely tuned to model varying injury severities, including mTBI (Siebold et al., 2018). Compared to FPI and weight-drop models, CCI provides superior control over injury biomechanics and avoids the risks of weight rebound or uncontrolled inertial forces, making it particularly valuable for mechanistic and translational TBI research (Xiong et al., 2013). Modern CCI devices include electromagnetic actuators that enhance reproducibility over pneumatic systems, and closed-head adaptations have been developed to better model concussion and mTBI without craniotomy. The model has also been widely used to investigate the effects of rmTBI. Despite its strengths, the CCI model primarily produces focal contusion-type injuries and is less suitable for modeling diffuse injury mechanisms characteristic of some forms of human concussion. Nevertheless, its scalability, reproducibility, and precise biomechanical control make CCI one of the most valuable and versatile models for experimental mTBI and rmTBI (Osier et al., 2017; Chahin et al., 2025).

## Microstructural, Neurochemical Alterations, and Connectivity Changes Following Mild Traumatic Brain Injury

**Figure 3:**
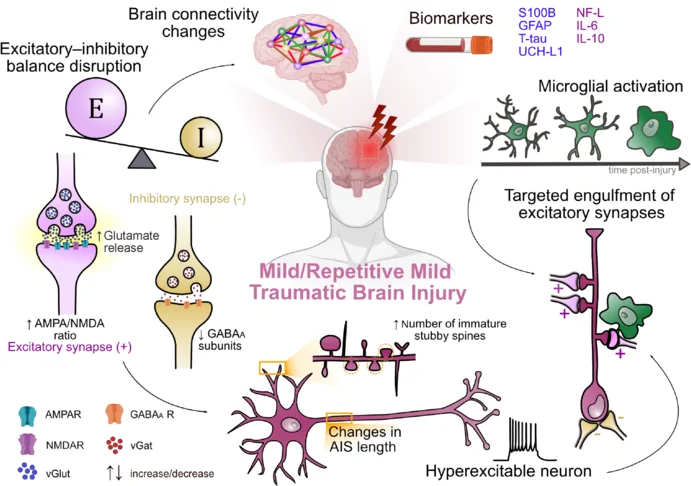
Schematic of the topics discussed in the review, including microstructural and connectivity changes, secondary injury, synaptic and microglia pathology, as well as biomarkers following mild traumatic brain injury and repetitive mild traumatic brain injury. Created with BioRender.com. ↑: Increase; ↓ Decrease. AMPAR: α-Amino-3-hydroxy-5-methyl-4-isoxazolepropionic acid receptor; GABA_A_ R: gamma-aminobutyric acid type A receptor; GFAP: glial fibrillary acidic protein; IL-6: interleukin-6; IL-10: interleukin-10; NF-L: neurofilament light chain; NMDAR: N-methyl-D-aspartate receptor; S100B: S100 calcium-binding protein B; T-tau: total tau; UCH-L1: ubiquitin C-terminal hydrolase L1; vGAT: vesicular GABA transporter; vGlut: vesicular glutamate transporter.

mTBI leaves a lasting neurobiological imprint. A single concussive event can disrupt large-scale brain networks within hours, while repeated impacts initiate cumulative cellular, vascular, and connective tissue alterations that progressively intensify with each additional insult (**Figure 3**).

The severity and specific characteristics of the resulting neurological deficits depend on both the location and extent of the injury (Bolandzadeh et al., 2012; Xu et al., 2021; de Oliveira et al., 2022). Recent advances in neuroimaging, particularly functional connectivity (FC) studies, have provided deeper insights into how these structural and microstructural brain changes relate to persistent functional and cognitive impairments in mTBI and rmTBI.

### Brain connectivity

FC studies have revealed widespread disruptions across multiple brain networks following mTBI. Affected networks include the frontoparietal, salience, default mode, dorsal attention, limbic, sensorimotor, and visual networks (**Table 1**; Sours et al., 2015; Madhavan et al., 2019; Champagne et al., 2020; España-Irla et al., 2025).

**Table 1:** Summary of studies investigating brain connectivity following mild and repetitive TBI

Single/Repetitive mild TBI	Species	Earliest time point reported post-injury	Affected networks	Brain regions within the networks	FC changes	Reference
Single	Human	5–18 d	Default mode network, frontoparietal network, salience network, dorsal attention network	Superior frontal cortex, inferior temporal cortex, and paracingulate gyrus, anterior cingulate gyrus	↓	España-Irla et al., 2025
		2 wk	Somatomotor network	Cerebellum and superior parietal pole		
			Visual network, dorsal attention network, frontoparietal network, limbic network	Occipital cortex, superior parietal lobule, supramarginal gyrus, temporal gyrus, frontal pole		
			Default mode network, visual network	Thalamus and occipital cortex, superior frontal cortex	↑	
Single	Human	Chronic: 6–60 mon	Dorsal attention network, somatomotor network	Superior parietal gyrus, precentral gyrus, supplementary motor area, primary somatosensory cortex	↑	Simos et al., 2023
Single	Human	Chronic: 1–48 mon	Frontoparietal network, default mode network	N/A	↓	Champagne et al., 2020
			Dorsal attention network, somatomotor network	N/A	↑	
Single	Human	7 d	Sensorimotor network	Cerebellum and sensorimotor cortex	↑	Vergara et al., 2018
Single	Human	24 h	Default mode network	Posterior cingulate cortex and medial prefrontal cortex	↓	Sours et al., 2015
		Chronic: 6 mon		Posterior cingulate cortex and task-positive regions	↑	
Single	Human	3 wk	Default mode network	Rostral anterior cingulate cortex and posterior cingulate cortex	↓	Mayer et al., 2011
Repetitive	Human	Chronic: 5 mon	Default mode network	Posterior cingulate cortex, precuneus, temporoparietal cortex, lateral temporal cortex, parahippocampal formation	↓	DeSimone et al., 2021
Repetitive	Human	72 h	Default mode network, limbic network, somatomotor network, salience network, visual network	Posterior cingulate cortex, left and right parahippocampal gyri, right cerebellum, anterior cingulate cortex, right fusiform gyrus	↓	Johnson et al., 2020
Repetitive	Human	1 mon	Default mode network	Posterior cingulate cortex, precuneus	↓	Abbas et al., 2015a, b
		2 mon			↑	
Repetitive	Human	Chronic: 3 wk–6 mon	Default mode network, salience network, executive network	Anterior cingulate cortex, dorsal anterior cingulate cortex, dorsolateral prefrontal cortex	↑	Czerniak et al., 2015

d: Day(s); FC: functional connectivity; h: hour(s); mon: month(s); N/A: not available; TBI: traumatic brain injury; wk: week(s); ↑: increase; ↓: decrease.

In a longitudinal investigation, Sours et al. (2015) observed reduced resting-state FC within the default mode network, particularly in the posterior cingulate cortex and medial prefrontal cortex, as early as 24 hours post-injury, with these deficits persisting up to six months. Supporting these findings, España-Irla et al. (2025), in a larger cohort, also reported default mode network hypoconnectivity with the frontoparietal, salience, and dorsal attention networks, highlighting the early and sustained vulnerability of higher-order cognitive networks to mTBI. Interestingly, Sours et al. (2015) also identified increased resting-state FC between the posterior cingulate cortex and task-positive regions during the chronic phase of mTBI, suggesting a compensatory strengthening of the default mode network and task-positive network integration over time.

In contrast to these cortical disruptions, Vergara et al. (2018) reported increased connectivity between the cerebellum and the sensorimotor network at 7 days post-injury. However, España-Irla et al. (2025) found reduced cerebellar connectivity with both the somatomotor and salience networks at 2 weeks post-injury. Follow-up analyses revealed that visual-motor performance, as measured by the Trail Making Test A, was significantly associated with a gradual normalization of cerebellar connectivity across widespread somatomotor regions over 12 months, suggesting functional reintegration of motor timing networks during recovery. Complementing these findings, Mayer et al. (2011) reported reduced FC between the rostral anterior cingulate cortex and the posterior cingulate cortex at 3 weeks post-injury, further evidencing early default mode network disruption.

Additionally, España-Irla et al. (2025) reported decreased FC between the visual network and the dorsal attention network, frontoparietal network, and limbic network. Notably, they also observed increased FC between the thalamus and both the occipital cortex and the superior frontal cortex, with these thalamo-cortical enhancements remaining stable from 2 weeks to 6 months post-injury. The authors interpreted these findings as a compensatory upregulation of thalamo-cortical relays, potentially serving to maintain the flow of sensory information despite diffuse injury. This pattern was also observed by Champagne et al. (2020), who reported both decreased and increased FC across cortical hubs, particularly within the default mode network, dorsal attention network, and frontoparietal network. Hypoconnectivity was primarily concentrated within the frontoparietal network and selected default mode network nodes, while, in contrast to what was reported by España-Irla et al. (2025), several dorsal attention network and somatomotor connections remained abnormally hypersynchronized relative to healthy controls. Increased FC within the dorsal attention network and somatomotor circuits was also observed by Simos et al. (2023), although their findings diverged by reporting increased FC within hubs of the default mode network. These variations in hyper- and hypoconnectivity likely reflect the heterogeneity of mTBI, which underlies the diverse symptom profiles observed across patients.

Hypoconnectivity in limbic and frontolimbic areas, while not directly correlated with anxiety or depression scores, may reflect subtler dysfunctions in emotion regulation, potentially contributing to symptoms such as irritability and impulsivity. Reduced blood-oxygenation-level-dependent signal complexity in the left anterior hippocampus also appeared to relate to diminished phonemic fluency. Despite the hippocampus being traditionally linked to episodic memory, its involvement in semantic processing tasks has been noted in cases of medial temporal amnesia (Greenberg et al., 2009; Sheldon and Moscovitch, 2012).

In rmTBI, similar FC alterations have been reported in the literature. Abbas et al. (2015) used head-impact telemetry with longitudinal rs-fMRI to detect changes in FC among high school football players over the course of a competition season, reporting hyperconnectivity within the default mode network. In a subsequent study conducted across two training seasons, the same group observed a more dynamic pattern: during the first month of the season, participants showed a significant decrease in default mode network connectivity, followed by an increase in the median number of default mode network connections during the second month, exceeding both the first-month and pre-season levels. The authors interpreted this as evidence of compensatory mechanisms in the brain, potentially activated to sustain normal behavior despite cumulative head impacts during the training season. Supporting this conclusion, Czerniak et al. (2015) reported increased FC within the default mode network, salience network, and executive control networks in a sample of athletes. Despite these increases, neurocognitive performance, assessed using the Wisconsin Card Sorting Task and the Color-Word Interference Test, did not differ significantly between athletes and healthy controls. The authors interpreted this pattern as evidence for the “working-harder” hypothesis, suggesting that increased metabolic or network-level recruitment may serve as a hidden cost of return-to-play, compensating for underlying injury.

In contrast, in more recent studies, both Johnson et al. (2020) and DeSimone et al. (2021) observed reduced default mode network connectivity in acute rmTBI cases among collegiate and youth football players, respectively. Johnson et al. (2020) conducted a single scan 72 hours post-injury, identifying significant reductions in FC between the posterior cingulate cortex (a key node of the default mode network) and multiple regions, including the left and right parahippocampal gyri, right cerebellum, anterior cingulate cortex, and the right fusiform gyrus. DeSimone et al. (2021), using a longitudinal design with pre-, post-, and off-season scans, found progressive decreases in default mode network connectivity across the football season, followed by a partial rebound during the off-season. This rebound was interpreted as evidence of neuronal recovery following the cessation of rmTBI exposure, potentially reflecting compensatory restoration of disrupted connectivity. Overall, these studies underscore both the damage endured by the brain following mTBI and rmTBI and the compensatory mechanisms engaged to maintain normal cognitive function.

### Microstructural brain alterations

With the aim of assessing the possible microstructural changes that drive these fluctuations in FC, several studies have used diffusion tensor imaging (DTI), neurite orientation dispersion and density imaging, positron emission tomography (PET), or a combination of these techniques (**Table 2**).

**Table 2:** Summary of studies investigating the microstructural brain alterations following mild and repetitive TBI

Single/Repetitive mild TBI, subjects	Species/Injury model	Time point post-injury	Technique	Affected hub	Changes	Reference
Single	Human	2 wk	DTI, NODDI	Genu and body of the corpus callosum, internal capsule, corona radiata, anterior thalamic radiation, external capsule, cingulum	↓ FA, NDI ↑ MD, FISO	Palacios et al., 2020
		6 mon		Posterior periventricular and left anterior periventricular WM	↓ NDI, FISO = FA, RD, MD, AD	
Single	Human	3 mon	MRI	Frontal lobe	WM hyperintensities	Patel et al., 2020
				Left transverse temporal gyrus, left insula, rostral anterior cingulate cortex, transverse temporal gyri, right lateral orbitofrontal cortex, right medial orbitofrontal cortex, right postcentral gyrus, right precentral gyrus, left cerebellar cortex, right thalamus	↓ Volume	
Single	Human	N/A	DTI	Amygdala-hippocampus complex, nucleus accumbens	↑ FA, MD	Sydnor et al., 2020
				Cingulate cortex	↓ FA	
				Left medial orbitofrontal cortex	↓ MD	
Single	Human	1 yr	DTI, NODDI	Frontal tracts: genu of corpus callosum, forceps minor, anterior thalamic radiation, cingulum, superior longitudinal fasciculus	↓ FA ↑ RD, MD, AD	Hellstrom et al., 2017
Single	Human	1 yr	MRI	Bilateral anterior cingulate WM, left cingulate gyrus isthmus WM, right precuneal GM	↓ Volume correlated with deficits in memory and attention	Zhou et al., 2013
Repetitive (×8)	Mouse CHI	7 d	Silver staining	Optic tract, corpus callosum, cortex	↑ Axonal injury	McDonagh et al., 2025
Repetitive (×2)	Mouse ACHI	5–6 d	rs-fMRI, DTI, histochemistry	Optic tract	↓ FA	Ravi et al., 2022
				Thalamus, visual network	Network disintegration	
				Optic tract, corpus callosum	Astrogliosis, microgliosis	
Repetitive (N/A)	Human	3 mon	DTI	Superior corona radiata	↑ FA	Stormezand et al., 2022
Repetitive (×20)	Mouse closed-head WD	45–90 d	Histochemistry	Optic tract	Astrogliosis	Angoa-Perez et al., 2020
		0, 45, 90 d			Microgliosis	
Repetitive (×2)	Rat FPI	1, 3, 5, 7, 30 d	DTI	Corpus callosum	↓ FA progressive ↑ RD	Wright et al., 2019
		30 d		Ipsilateral hemisphere	Marked cortical tissue loss	

ACHI: Accelerated closed-head injury; AD: axial diffusivity; CHI: closed-head injury; d: day(s); DTI: diffusion tensor imaging; FA: fractional anisotropy; FISO: isotropic volume fraction; FPI: fluid percussion injury; GM: gray matter; h: hour(s); MD: mean diffusivity; mon: month(s); MRI: magnetic resonance imaging; N/A: not available; NDI: neurite density index; NODDI: neurite orientation dispersion and density imaging; RD: radial diffusivity; rs-fMRI: resting-state functional magnetic resonance imaging; TBI: traumatic brain injury; WD: weight drop; wk: week(s); WM: white matter; =: unchanged parameter; ↑: increase; ↓: decrease.

Zhou et al. (2013) performed MRI scans in patients with mTBI at 23 days and 1 year post injury, observing significant global brain atrophy at one-year post injury, including volume reductions in bilateral anterior cingulate white matter, the left cingulate gyrus isthmus white matter, and the right precuneal gray matter. Notably, volume loss in the left and right rostral anterior cingulate white matter was strongly correlated with deficits in memory and attention, suggesting a functional impact of these structural changes. Reinforcing the notion that brain atrophy is not exclusive to moderate or severe TBI, these findings demonstrate that measurable structural degeneration can also occur following a single mTBI.

In a DTI and neurite orientation dispersion and density imaging study involving more than 100 mTBI patients, Hellstrom et al. (2017) reported a correlation between increased symptoms and widespread reductions in fractional anisotropy and increases in radial, mean, and axial diffusivity. These alterations were observed 12 months after injury, being stronger in the frontal tracts, including the genu of the corpus callosum, forceps minor, anterior thalamic radiation, cingulum, and superior longitudinal fasciculus. These findings point to post-injury long-term axonal and myelin degradation, likely contributing to the persistence of cognitive, somatic, and emotional symptoms. Similarly, in a longitudinal study, Palacios et al. (2020) reported an acute decrease in fractional anisotropy and an increase in mean diffusivity, accompanied by elevated free-water fraction, particularly in the genu and body of the corpus callosum, internal capsules, corona radiata, anterior thalamic radiation, external capsule, and cingulum. At six-month follow-up, they observed a reduction in neurite density index, predominantly in the posterior periventricular and left anterior periventricular white matter, while fractional anisotropy, radial diffusivity, mean diffusivity, and axial diffusivity remained largely unchanged. The authors interpreted these findings as indicative of acute edema, with the subsequent decline in neurite density index suggesting ongoing axonal degeneration.

Using MRI, Patel et al. (2020) reported white matter hyperintensities, particularly in the frontal lobe of symptomatic military personnel 3 months after mTBI, additionally reporting decreased volume in the left transverse temporal gyrus. Furthermore, the authors reported decreased volume of certain gray matter regions, such as the left insula, rostral anterior cingulate cortex, transverse temporal gyri, right lateral orbitofrontal cortex, right medial orbitofrontal cortex, right postcentral gyrus, and right precentral gyrus, as well as subcortical gray matter regions such as the left cerebellar cortex and the right thalamus.

Additional evidence points to microstructural alterations in the right cingulate cortex. In individuals with comorbid mTBI and PTSD, Sydnor et al. (2020) reported that greater PTSD symptom severity was associated with diffusion abnormalities, including increased fractional anisotropy in the amygdala–hippocampus complex and nucleus accumbens, as well as decreased fractional anisotropy in the cingulate cortex, and elevated mean diffusivity in the right amygdala–hippocampus region but lower mean diffusivity in the left medial orbitofrontal cortex. These findings suggest that mTBI may amplify the impact of structural brain changes on PTSD symptom expression.

Decreased volume in gray matter regions, along with reduced FC in the associated networks, provides a compelling neurobiological framework for the persistent mood disturbances, interoceptive symptoms, and executive dysfunction frequently observed in mTBI patients. These regions represent key cortical hubs within the salience, default mode, and reward networks, and their degeneration likely disrupts the dynamic integration required for adaptive emotional and cognitive functioning (Seeley et al., 2007; Menon, 2011; Rolls, 2019). Furthermore, evidence of atrophy in subcortical gray matter structures suggests that mTBI is not confined to superficial cortical areas but also affects deep integrative hubs essential for large-scale brain network coordination.

Research on microstructural brain changes in human rmTBI is still scarce. However, a recent study by Stormezand et al. (2022) examined active elite kickboxers and provided preliminary evidence of microstructural alterations associated with rmTBI. Using DTI, the authors reported increased fractional anisotropy bilaterally in the superior corona radiata, an observation that contrasts with the decreased fractional anisotropy reported previously in mTBI subjects (Palacios et al., 2020). Given the small sample size, these findings should be interpreted with caution and require replication in larger cohorts to confirm their reliability.

However, multiple animal studies have demonstrated that rmTBI leads to persistent, region-specific white matter abnormalities. Using diffusion-weighted MRI in a FPI rmTBI rat model, Wright et al. (2019) reported reduced fractional anisotropy in the ipsilateral corpus callosum, indicative of axonal damage, and increased radial diffusivity, suggesting progressive compression or disorganization of axonal fibers across multiple post-injury time points (days 1, 3, 5, 7, and 30). In addition to white matter changes, the authors observed cortical abnormalities in the ipsilateral hemisphere, including visible deformation and midline shift, which progressed to marked cortical tissue loss by day 30 post-injury.

Angoa-Perez et al. (2020) similarly reported evidence of axonal damage, though in a different species model, a mouse weight drop closed-head rmTBI paradigm. The study evaluated glial activation and recognition memory at 0, 45, and 90 days post-injury. Findings revealed pronounced white matter vulnerability, characterized by progressive optic tract astrogliosis beginning at day 45 and increasing through day 90, alongside persistent microgliosis observed at all time points. These results suggest early microglial priming, followed by sustained astrocytic reactivity. The progression of glial responses was paralleled by declines in recognition memory, supporting the interpretation of a developing CTE-like white matter disease trajectory.

Similarly, both Ravi et al. (2022) and McDonagh et al. (2025) reported axonal pathology by evaluating optic tract integrity in rmTBI mouse models. Ravi et al. (2022) employed a closed-head impact model of engineered rotational acceleration combined with rs-fMRI and DTI, identifying decreased fractional anisotropy in the optic tract and early network disintegration involving thalamic and visual hubs. They also reported pronounced astro- and microgliosis in the optic tract and corpus callosum, implicating glial activation in the progression of axonal damage. McDonagh et al. (2025) used an advanced closed head injury model in both male and female mice, combining silver staining with cognitive assessments. Their results showed increased axonal injury, evidenced by elevated silver staining in the optic tract, corpus callosum, and cortex at 7 days post-injury, primarily driven by female subjects. Interestingly, these structural changes were not accompanied by measurable cognitive impairments, suggesting that sex-specific diffuse axonal injury may occur in the absence of overt behavioral deficits.

Overall, mTBI and rmTBI lead to measurable, often progressive, microstructural and volumetric brain changes that disrupt critical neural networks, resulting in persistent cognitive, emotional, and behavioral symptoms. These changes are evident across species and are influenced by factors such as sex and comorbid conditions such as PTSD. The findings underscore the importance of considering both superficial and deep brain structures in understanding the long-term consequences of traumatic brain injury.

### Neurochemical brain alterations

In addition to connectivity and microstructural alterations, rmTBI has been shown to disrupt neurotransmitter systems. Horvat et al. (2024) examined how rmTBI disrupts norepinephrine signaling. Male and female rats received either a single CCI or three identical impacts 24 hours apart. A microdialysis probe placed in layer V of the prelimbic medial prefrontal cortex measured basal norepinephrine (NE) efflux and the response to methylphenidate (MPH), a stimulant known to increase NE availability. After one impact, both sexes showed normal basal NE levels and a normal NE surge after MPH. After three impacts, the medial prefrontal cortex entered a hypoadrenergic state, and active-phase waking (the period when rats are typically awake) was shortened. When challenged with MPH, males, but not females, failed to elevate NE. The higher basal NE seen in females emerged only after the repeated-impact condition, and females retained a normal NE response to MPH. The authors concluded that repetitive, but not single, mild head injury causes an early deficit in locus-coeruleus–mediated NE signaling that may drive cognitive fatigue and sleep–wake disturbance after multiple concussions, with males showing greater pharmacological insensitivity to psychostimulants.

In addition to disruptions in the NE system, alterations in dopaminergic signaling have also been observed following mTBI. Using the Marmarou weight-drop impact-acceleration model, Wee et al. (2024) examined both mTBI and rmTBI conditions (moderate-to-severe TBI data were excluded as they fall outside the scope of this review). The authors reported an approximately 80% increase in dopamine D1 receptor expression in the prefrontal cortex following a single mTBI, with no additional changes observed in the rmTBI group. This upregulation was interpreted as a receptor-level compensatory response to transient dopaminergic fluctuations induced by mild injury. Together, these results underscore the complex neurochemical consequences of mTBI and rmTBI, highlighting their potential to drive cognitive alterations.

Adding to the reported alterations, rmTBI can also compromise the glymphatic system, which plays a critical role in clearing metabolic waste from the brain. Christensen et al. (2020) employed a rat lateral-impact rotational-acceleration mTBI model in which a pneumatic pressure-propelled weight struck the side of the head, causing the animal to spin 180° horizontally, a less commonly used mTBI paradigm. Using contrast-enhanced MRI, the authors found increased tracer influx but reduced efflux in limbic regions and the olfactory bulb, while no significant changes were observed in cortical influx or efflux. They proposed that impaired clearance in limbic structures may contribute to the development of post-concussive symptoms. Supporting these findings, Michalaki et al. (2025) used a closed-head CCI rmTBI model combined with near-infrared imaging, reporting reduced near-infrared signal and slower dye clearance in the lateral ventricle and adjacent parietal cortex, findings consistent with decreased cerebrospinal fluid perfusion following rmTBI.

## Secondary Injury following Mild Traumatic Brain Injury and Repetitive Mild Traumatic Brain Injury

Secondary brain injury following mTBI and rmTBI is driven by a complex array of pathological mechanisms, including excitotoxicity, oxidative stress, inflammation, mitochondrial dysfunction, disruption of the BBB, and metabolic imbalance, ultimately resulting in neurological impairment (**Figure 3**).

### Inflammatory responses

Inflammation is a known part of the secondary injury in TBI (Gonzalez et al., 2016; Freire et al., 2023). In recent years, the inflammatory profile of mTBI and rmTBI has been better described. Nespoli et al. (2024) employed a weight-drop closed-head mTBI mouse model and used BrdU proliferation assays alongside immunohistochemistry for Iba1, NG2, and glial fibrillary acidic protein (GFAP) to characterize glial responses following mTBI. The authors reported a limited response from microglia and NG2-positive glia within the first 3 days post-injury, whereas astrocytic proliferation peaked between 3 and 7 days, indicating a delayed but robust astrocytic activation phase.

In a related study, Drieu et al. (2022) used a closed-head CCI mTBI mouse model combined with MRI and a behavioral test battery to assess injury outcomes. Despite the absence of overt contusions, mice exhibited persistent cognitive and sensorimotor deficits. Translocator protein (TSPO)-PET imaging revealed chronic gliosis in the cortex and hippocampus lasting up to three weeks post-injury, highlighting the presence of sustained neuroinflammation even in the context of mild structural trauma. There was a distinct systemic inflammatory response detectable from 6 hours until 1 year after mTBI, including expression of interleukin (IL)-6 and IL-10, underscoring the delayed and persistent nature of neuroinflammation following mTBI (Visser et al., 2022).

As in mTBI, neuroinflammation and microglial priming play a central role in the pathology of rmTBI. As previously discussed in this review, Hiskens et al. (2021) employed a weight-drop closed-head rmTBI model (1, 5, and 15 hits over 23 days), combining quantitative real-time polymerase chain reaction, serum GFAP/p-tau analysis, and the Morris water maze to evaluate molecular and behavioral outcomes. The study found that rmTBI impaired spatial memory and was associated with upregulation of key excitotoxicity- and inflammation-related genes in the cortex and hippocampus, including *MAPT*, *GFAP*, *AIF1*, *GRIA1*, *CCL11*, *TARDBP*, *TNF*, and *NEFL*. These findings support a dose-dependent relationship between repetitive impacts, sustained cognitive deficits, and glial/inflammatory gene activation.

Complementing these findings, Wu et al. (2019) used a closed-head rmTBI mouse model and behavioral assessments alongside IL-1β and IL-18 immunostaining to investigate the neuroimmune response. Their results demonstrated that three daily impacts triggered the maturation of endothelial IL-1β and IL-18, facilitating inflammasome signaling that contributed to learning impairments. While Hiskens et al. (2021) characterized the molecular signature of chronic neuroinflammation, Wu et al. (2022) highlighted its causal role in symptom development and pointed toward inflammatory signalling as a potential therapeutic target.

In a subsequent study, Wu et al. (2022) used the same rmTBI paradigm as before and single-nucleus RNA sequencing, neuronal Westerns, IL-1R1 knockout, and behavioral tests to identify the disruption of genes linked to neuronal proteostasis, while western blots showed inflammasome activation and hyperphosphorylated tau at 14 months. The IL-1R1 knockout reduced pro-IL-1β and misfolded tau. Interestingly, males showed specific tauopathy, while females had anxiety but no tau buildup.

Building on this inflammation-centric picture, Bielanin et al. (2024) employed a closed-skull controlled-concussion paradigm (five impacts, 48-hour interval) and focused on the Na/H exchanger 1 (NHE1) as an upstream regulator of glial activation. They observed that rmTBI upregulated NHE1 in GFAP astrocytes, Iba-1 microglia, and OLIG2 oligodendrocyte-lineage cells, coincident with oxidative stress, gliosis, β-amyloid precursor protein accumulation, and callosal white matter damage. Remarkably, post-injury treatment with the NHE1 inhibitor HOE642, started 24 hours after the final concussion, rescued motor learning, improved spatial memory, and blunted glial/oxidative pathology.

Systemic inflammatory responses and inflammasome signaling contribute to cognitive and behavioral deficits, with repetitive injury amplifying these effects. Targeting neuroinflammation and related oxidative stress pathways offers promising therapeutic avenues for mitigating the long-term consequences of mTBI and rmTBI.

### Vascular compromise: Blood–brain barrier disruption

The BBB is a set of cellular and tissue layers that form a semipermeable barrier whose function is to control the influx and efflux of molecules and cells in the CNS, being able to exclude approximately 98% of all small-sized drugs (Pandit et al., 2020). Recent research has increasingly identified the BBB as an early and dynamic target of disruption following mTBI, even in the absence of classical histopathological changes.

Wu et al. (2020), in a comprehensive review of the murine literature spanning two decades, highlighted that BBB permeability often emerges within 15 to 60 minutes post-injury and can precede or even occur without overt neuroinflammatory or structural damage. This challenges traditional diagnostic frameworks that rely on observable neuronal loss or gliosis, instead positioning vascular integrity as an independent and sensitive index of brain trauma. Supporting this, Hiles-Murison et al. (2021) provided experimental evidence using a weight-drop sub-concussive rmTBI rat model, reporting significant BBB leakage and ventricular enlargement, despite the absence of gliosis or excessive neurobehavioral impairment. Together, this suggests that BBB breakdown may serve as a silent but measurable indicator of neuropathological change, and that vascular compromise may underlie a latent phase of injury not captured by conventional assessments.

Further insight into the relationship between vascular dysfunction and mTBI comes from Wu et al. (2019), who used a closed-head weight-drop rmTBI model to investigate the role of endothelial inflammasome activation. In a model of three daily impacts in adolescent mice, they showed that processed IL-1β was present in wild-type brain endothelium and linked this activation to cognitive deficits. These impairments were absent in IL-1R1 and caspase-1 knockout mice, suggesting a key role for IL-1 signaling in mediating the effects of injury. This work aligns with the vascular emphasis seen in Hiles-Murison et al. (2021), but offers a different angle, pointing to cytokine-driven dysfunction rather than structural damage as the primary trigger for symptoms.

Logsdon et al. (2018) provide yet another layer to this evolving picture, highlighting the role of nitric oxide in BBB permeability. Using a mild blast rmTBI mouse model, they reported that two hits were enough to provoke biphasic BBB opening, with delayed disruption of the tight junction protein claudin-5. Importantly, this effect was blocked by L-NAME, a nitric oxide synthase inhibitor, suggesting that tight junction integrity is actively regulated and can be protected pharmacologically. Although Logsdon et al.’s findings focus on nitric oxide and tight junction disruption, both Wu et al. (2020) and Logsdon et al. (2018) underscore that BBB dysfunction in mild TBI is not a fixed event, but rather a dynamic process that can evolve through multiple mechanisms over time. Finally, mTBI reduces cerebral blood flow (Kagialis et al., 2024), which, coupled with increased energy demands, leads to a metabolic crisis, the severity of which varies depending on the extent of injury (Giri et al., 2000; Fehily and Fitzgerald, 2017). As the brain primarily relies on glucose (Cacciatore et al., 2022), it is particularly susceptible to such disturbances, causing hypoglycemia, cognitive decline (Komura et al., 2019), and insulin resistance (Franklin et al., 2019). These alterations impair cerebral function and worsen clinical outcomes (Lai et al., 2022).

### Excitotoxicity and ionic imbalance

Excitotoxicity refers to the pathological process by which excessive glutamate release or overstimulation of glutamate receptors results in neuronal injury or death (Neves et al., 2023). While this mechanism is well established in moderate to severe TBI (Kaur and Sharma, 2018), it is less extensively characterized in the context of mTBI and rmTBI. Nonetheless, recent evidence has investigated glutamate release dynamics following mild and repetitive injury.

Masse et al. (2024) employed an awake rat model of single and repeated weight-drop rotational mTBI combined with continuous hippocampal microdialysis to assess post-injury neurotransmitter dynamics. Following the first injury, there was a substantial increase in extracellular levels of glutamate, GABA, taurine, and glycine. However, the second injury (rmTBI), administered 24 hours later, elicited a markedly attenuated neurotransmitter response. The authors interpreted this blunted effect as indicative of metabolic exhaustion or the development of adaptive tolerance when concussions occur in rapid succession.

Glutamatergic alterations have also been reported in other mTBI models. Garcia et al. (2025) used a CCI model with parameters consistent with mTBI and employed real-time glutamate imaging alongside immunohistochemistry to assess glutamate dynamics at 3, 7, and 14 days post-injury. The authors observed elevated glutamate release at 3 days post-injury, accompanied by reduced astrocytic glutamate transporter-1 expression, suggesting impaired glutamate reuptake. Both measures returned to baseline by days 7 and 14, indicating a transient disruption of glutamate clearance. In contrast, Talty et al. (2024), using a closed-head CCI mTBI model, reported persistent downregulation of glutamate transporter-1 in the hippocampus from 8 to 12 weeks post-injury, along with increased expression of NMDA receptor subunits, including GluN1, in the frontal cortex and amygdala. These findings point to chronic glutamatergic dysregulation, marked by reduced reuptake and enhanced excitatory signaling in mood-relevant circuits, an imbalance that may underlie the impaired self-care and social behaviors observed at chronic time points.

Excitotoxicity has also been reported in rmTBI. Hiskens et al. (2021) used a weight-drop rmTBI mouse model that received either 1, 5, or 15 blows, along with mRNA analysis and Morris water maze testing, to show that 15 impacts impaired spatial memory and upregulated excitotoxic (GRIA1) and inflammatory (TNF, among others) genes in both the cortex and hippocampus. This was interpreted by the authors as a window of cerebral vulnerability, where closely spaced impacts amplify molecular cascades. Supporting these findings, Allen et al. (2023) employed an awake closed-head mTBI and rmTBI rat model in combination with proton magnetic resonance spectroscopy to assess metabolic alterations. Twenty-four hours after the final injury, animals in the rmTBI group exhibited elevated hippocampal glutamine levels and reduced glucose concentrations, whereas a single mTBI produced no detectable metabolic changes. Additionally, sensorimotor deficits were observed only within the first 1 to 4 hours post-injury, highlighting the transient nature of acute functional impairments in contrast to the more persistent metabolic disruptions seen following repeated injuries. Overall, these studies show that multiple mild impacts to the head lead to harmful processes such as excitotoxicity and inflammation, as well as persistent disruptions in brain metabolism. While the immediate symptoms of injury, such as sensorimotor deficits, may be short-lived, these underlying molecular and metabolic changes can create a period of heightened vulnerability and may contribute to long-term cognitive and behavioral problems. This underscores the importance of preventing repeated head injuries and monitoring individuals for both acute and delayed consequences.

### Oxidative stress and mitochondrial dysfunction

Oxidative stress, an imbalance between reactive oxygen/nitrogen species and antioxidant defenses, has been implicated in the pathophysiology of mTBI (Ismail et al., 2020). Petronilho et al. (2010) utilized a Marmarou mTBI rat model to assess oxidative damage, using thiobarbituric acid reactive substances as an index of lipid peroxidation and protein carbonylation as a marker of protein oxidation. The authors reported increased protein oxidation in the cerebellum at both 30 minutes and 6 hours post-injury. In the cortex, protein carbonyl levels were elevated at all assessed time points, while in the hippocampus, increases were noted at 3 and 6 hours post-injury. In the striatum, significant elevations in protein carbonylation were observed as early as 30 minutes and persisted through 3 and 6 hours.

Mitochondria, the vital organelles responsible for cellular energy production, are highly vulnerable to both mTBI and rmTBI. Ismail et al. (2020) showed that mitochondrial reactive oxygen species production increases post-TBI, causing oxidative stress. In a recent study using a weight-drop mouse model, Mira et al. (2023) examined early mitochondrial responses to mTBI through calcium uptake assays and electron microscopy. They found that, within 24 hours of injury, hippocampal mitochondria exhibited a reduction in membrane potential and increased basal matrix calcium levels. These changes were accompanied by elevated mitochondrial calcium efflux, stable calcium uptake, and increased expression of the mitochondrial Na^+^/Ca²^+^ exchanger protein NCLX, suggesting that hippocampal cells upregulate NCLX to compensate for calcium overload and help restore mitochondrial homeostasis. Complementing these findings, Allen et al. (2023) employed an awake, closed-head mTBI and rmTBI rat model and used *in vivo* ^1^H-MRS to assess metabolic and behavioral outcomes. Their results revealed that significant metabolic disturbances, including cortical lipid alterations, increased hippocampal glutamine, and decreased hippocampal glucose, as well as behavioral deficits, were largely confined to the rmTBI group, highlighting the heightened vulnerability and broader dysfunction associated with repeated injury. Together, these studies demonstrate that both single and repeated mild brain injuries disrupt mitochondrial function, but repetitive injury leads to more pronounced metabolic and behavioral consequences.

Similarly, Bielanin et al. (2024) used a closed-head CCI rmTBI mouse model to report an elevation of the NHE1 protein, accompanied by astrogliosis, microgliosis, and β-amyloid precursor protein accumulation, which the authors interpreted as a pH-sensitive trigger of reactive oxygen species production and glial activation. Additionally, Wright et al. (2019) used a fluid percussion rmTBI rat model and serial structural, diffusion MRI, proteomics, and sensorimotor tests, reporting abnormalities in the cortex and corpus callosum. Proteomic analysis of plasma identified markers indicating axonal and vascular injury, as well as metabolic and mitochondrial dysfunction and glial reactivity. All these changes were paired with cognitive and sensorimotor deficits.

Impaired mitochondrial respiration was also reported by Hubbard et al. (2023) in synaptic mitochondria isolated from the prefrontal cortex and the amygdala/entorhinal/piriform cortex region using a blast-induced rmTBI model. Interestingly, increased oxidative damage was also observed in mitochondria from glial cells, while no significant oxidative damage was detected in synaptic mitochondria, suggesting cell-type-specific vulnerability of mitochondrial populations to blast-induced injury.

Together, these studies convey that mTBI and rmTBI disrupt mitochondrial function, leading to calcium dysregulation, metabolic disturbances, and behavioral deficits. Repeated injury amplifies these effects, causing more severe and widespread dysfunction. The response is not uniform across all cell types, with glial mitochondria being more susceptible to oxidative damage than synaptic mitochondria.

### Astrogliosis

In addition to microglia, astrocytes are a major type of glial cells that play a crucial role in the brain’s response to TBI. They interact with microglia and other cells to coordinate this response and undergo rapid functional and molecular changes following injury, including altered gene expression and morphology. These changes are triggered by mechanical and biochemical signals such as calcium influx, extracellular adenosine triphosphate (ATP), and damage-associated molecular patterns. In cases of mild reactive astrogliosis, astrocytes show subtle morphological alterations, including swollen, thickened primary processes and reduced complexity of fine branches. GFAP-positive hypertrophic astrocytes appear in scattered clusters within the cortical gray matter, typically without widespread proliferation or glial scar formation (Shandra et al., 2019).

The astrocyte response, however, varies with injury type and developmental stage. In repetitive mild TBI models using adult mice, astrocytes display a non-classical response marked by rapid and sustained loss of key homeostatic proteins such as glutamate transporter-1, Kir4.1, Connexin 43, glutamine synthetase, and S100β. This atypical phenotype, which occurs without hypertrophy or proliferation, is linked to BBB disruption and persists for months, suggesting impaired astrocyte function and a potential role in long-term neurological deficits, such as cognitive decline, mood disturbances, and epilepsy (Shandra et al., 2019; Muñoz-Ballester et al., 2023).

In contrast, findings from Clément et al. (2020), using juvenile mice subjected to a single mild TBI, described a more classical form of reactive astrogliosis. Astrocytes in this model exhibit increased GFAP expression and hypertrophic morphology near the impact site, with these changes progressively spreading to distant brain regions. Despite the absence of astrocyte proliferation in both models, astrocytes in the juvenile brain retain core protein expression and structural complexity, suggesting a more adaptive or compensatory role. Overall, while repetitive mTBI induces a potentially pathological astrocyte phenotype, single mild TBI in the developing brain elicits a more graded and spatially evolving astrocytic response.

### Cell death

Whether mTBI induces cell death remains a topic of debate due to its typically low-severity damage. Some preclinical experimental models involving rmTBI (e.g., five CCI impacts administered at 24-hour intervals) have reported significant acute neuronal death in regions such as the entorhinal cortex and around hemorrhagic lesions (Bolton and Saatman, 2014). However, similar models with different injury intervals (e.g., 48 hours) did not show comparable neurodegenerative changes (Luo et al., 2014). This variability suggests that cumulative injury burden and inter-injury timing and grade may influence outcomes. Despite the lack of overt neuronal loss in many cases, functional impairments and diffuse axonal injury are believed to underlie the chronic progression of mTBI. Glial cell death in rmTBI remains relatively understudied.

## Microglial Changes in Response to Mild Traumatic Brain Injury

Microglial cells serve as resident immune cells in the central nervous system, playing essential roles in maintaining neural function. They support neurons through synaptic pruning, surveillance of neural activity, and defense against injury or infection. Even under normal conditions, microglia remain highly dynamic, constantly extending and retracting their processes as they actively monitor the brain environment. In response to injury, microglia detect extracellular signals that trigger rapid migration to the damaged site, where they contribute to the formation of a protective barrier that isolates injured tissue from surrounding healthy regions (Davalos et al., 2005; Dou et al., 2012). This activation results in immediate morphological and functional changes that persist for weeks to months. While such responses have been well documented in more severe TBI models, similar alterations are also observed following mild traumatic brain injury, indicating that even subtle impacts can induce sustained microglial reactivity (**Figure 3** and **Additional Table 1**).

**Additional Table 1:** Summary of papers investigating structural and functional microglial changes following mTBI and rmTBI between 2014 and 2025

Injury model	Single/repetitive mild TBI	Species	Time points post-injury	Brain regions	Microglial functional activation	Microglial structural activation	Microglia density	Reference
CHIMERA	Single	Mouse	7 d	Neocortex, thalamus, DG, optic tract	↑ Activation/microgliosis; ↑ Additional glial activation signatures in the cortex and thalamus	N/A	N/A	Swaro et al., 2025
CCI	Single	Mouse	7, 10, 14, 42 d	Sensorimotor cortex	↑ Microglial activation; ↑ Phagocytosis of GFP^+^ neuronal debris	N/A	Microgliosis peaks at ~10 dpi and resolves by 42 dpi	Alkaslasi et al., 2025
WD	Single	Mouse	1, 3, 7, 15 d	Cortex	↑ Activation (degree III, 3 d); ↑↑ Activation (degree IV, 3-15 d)	↑ Hypertrophic, activated (degree III, 3 d); ↑↑ Amoeboid, highly activated (degree IV, 3 to 15d)	↑ Microglial number (degree III, 3 d); ↑↑ Microglial number (degree IV, 3-15 d)	Nespoli et al., 2024
Closed-head rotational acceleration	Single	Pig	3, 7, 30 d, 1 yr	Medial cortex (cingulate gyrus) and lateral cortex (temporal gyrus)	↑ Microglial activation (acute in temporal gyrus, 3-7 d; chronic in cingulate, 30 d-1 yr)	Hyper-ramified ↑ branches, junctions, endpoints, process length (30 dpi → 1 ypi)	N/A	Grovola et al., 2023
CCI	Single	Mouse	1, 7, 21 d	Cortex, hippocampus	↑ Activated microglia	N/A	↑ Total microglia (ipsilateral)	Drieu et al., 2022
CFP	Single	Pig	1 min, 30 min, 3 h, and 6 h	Thalamus	↑ Activation (6 h post-injury)	↓ Cell area, thickened and shortened processes (6 h)	N/A	Lafrenaye et al., 2020
Modified WD	Single	Mouse	1-48 h	Piriform and entorhinal cortices	↑ Microglial activation	Swollen somata and thickened processes; microglial clusters	No change	Witkowski et al., 2019
CCI	Single	Mouse	6 h, 1-7 d, 3 mon	Cortex	Activation markers: ↑↑ pro-inflammatory (6h-1d), ↑ anti-inflammatory at 2-3 d	N/A	N/A	Taib et al., 2017
FPI	Single	Rat	7, 28 d	Primary somatosensory barrel field, ventral posteromedial nucleus	↑ Microglial activation	Swollen cell bodies and fewer, thicker, shortened processes	↑ Microglial proliferation	Cao et al., 2012
WD	Repetitive (x3)	Mouse	5, 7 d	Cortex	↑ Microglial activation; ↓ activation 7 d	↑ Hypertrophy/increased soma size (5 d)	N/A	Kaiser et al., 2025
CHIMERA	Repetitive (x3)	Rat	4 d	Primary somatosensory cortex	↑ Mild activation	Ameboid form	N/A	Boese et al., 2024
ACHI	Repetitive (x8)	Mouse	1, 3 d	Cortex, hippocampus (DG), midbrain regions	N/A	↑ Activated morphology at 1 d; returns to resting at 3 d	↑ Microglial proliferation, returns to baseline by 3 d	Neale et al., 2023
CCI	Repetitive (x3)	Mouse	1 wk	Cortex, hippocampus	N/A	Ramification and elongation	N/A	Schwab et al., 2022
CCI	Repetitive (x5)	Mouse	8-48 h; 2, 6, 10, 16 wk	Prefrontal cortex, corpus callosum, hippocampus	↑ Microglial activation (peaks at 8-48h); resolves by 1wk	Activated rounded, bushy morphology (48 h); resting baseline appearance by 1 wk	N/A	Xu et al., 2021
CHI	Repetitive (x7);	Mouse	24 h, 1 wk	Fimbria, hippocampus	↑ Microglial activation	N/A	↑ Microglia density	Robinson et al., 2017
CCI	Repetitive (x2)	Mouse	2, 4, 7, 14, 28, 49 d	Corpus callosum, cortex, thalamus, hippocampus	↑ Microglial activation	Hypertrophic, bushy, and amoeboid morphologies	↑ Microglia density	Shitaka et al., 2011
CCI	Single/repetitive (x3)	Mouse	48 h, 1 wk, 3 wk, 6 wk	Sensorimotor cortex (layer II/III) and hippocampus (CA2, CA3, DG)	Single: transient microglial activation and excitatory synapse engulfment Repetitive: robust, prolonged microglial activation and excitatory synapse engulfment	Single: transient hypertrophy Repetitive: persistent morphological activation	None after single and repetitive	Chahin et al., 2025
WD	Single/repetitive (x2, x3)	Rat	2 wk, 4 wk	Prefrontal cortex, corpus callosum, hippocampus (CA1)	Microglial activation: ↑ 2wk in PFC, corpus callosum, CA1	Single and repetitive (x2): little to no changes Repetitive (x3): retracted processes	Single and repetitive (x2): transient ↑ Repetitive (x3): ↑ microglial density	Sugahara et al., 2025
Air blast	Single/repetitive (x4)	Mouse	4, 15 mon	Hippocampus	Single: ↑ microglia activation Repetitive: ↓ microglia activation	Repetitive: ↓ soma volume, soma roundness, process length, branches, and complexity	Repetitive: ↓ microglia density or no change	Honig et al., 2021
CHI	Single/repetitive (x2 or x3)	Rat	3d, 1 wk	Trigeminal nucleus caudalis	Single: little to no activation Repetitive: ↑ Activated, proliferating microglia	Single: no changes Repetitive: thickened and retracted processes	Single: ↑ microglia density Repetitive: ↑↑ microglia density	Tyburski et al., 2017
LFP	Single/repetitive (x3)	Rat	28 d	Cortex, hippocampus	Single: moderate microglial activation ipsilateral cortex; no significant activation in the hippocampus Repetitive: strong ↑ in microglial activation	Repetitive: hypertrophic and bushy appearance	Repetitive: ↑ microglia density	Aungst et al., 2014

Summary of papers investigating structural and functional microglial changes following mTBI and rmTBI between 2014 and 2025. ACHI: Accelerated closed-head injury; CA1, CA2, CA3: cornu ammonis regions 1, 2, and 3 of the hippocampus; CCI: controlled cortical impact; CFP: central fluid percussion; CHI: closed-head injury; CHIMERA: closed-head impact model of engineered rotational acceleration; d: day(s); DG: dentate gyrus; dpi: days post-injury; FPI: fluid percussion injury; GFP: green fluorescent protein; h: hour(s); Iba-1: ionized calcium-binding adapter molecule 1; LFP: lateral fluid percussion; min: minutes; mon: month(s); mTBI: mild traumatic brain injury; rmTBI: repetitive mild traumatic brain injury; N/A: not available; PFC: prefrontal cortex; wk: week(s); WD: weight drop; ypi: years post-injury; yr: year(s); ↑: increase/upregulation; ↑↑: strong increase/marked upregulation; ↓: decrease/downregulation; →: transformation or transition.

### Structural and functional activation

Microglial activation emerges rapidly following single mild TBI, with detectable changes within hours. For instance, Lafrenaye et al. (2020) reported that microglia in the cortex and hippocampus displayed an activated phenotype as early as 6 hours following a mild central fluid percussion injury in micropigs. Interestingly, unlike astrocytic markers such as GFAP, which rise in peripheral blood after injury, microglial markers such as Iba-1 do not exhibit significant acute changes in serum following mild TBI. This likely reflects microglia’s residency within the CNS and the limited permeability of the blood-brain barrier during the early post-injury phase, which restricts microglia-derived proteins from entering peripheral circulation. These early changes are accompanied by morphological shifts, including hypertrophy and process retraction, that signal microglial activation. Interestingly, in larger animal models such as pigs subjected to rapid head rotation in the coronal plane to generate mild TBI, microglia exhibit a distinct hyper-ramified morphology with increased branching that persists long-term, suggesting species- and injury-specific differences in microglial responses (Grovola et al., 2023).

In mouse CCI models, microglial activation extends beyond the acute phase. Drieu et al. (2022) reported sustained increases in activated microglia (Iba1^+^/CD68^+^) in the cortex and hippocampus lasting up to 21 days post-injury following closed-head controlled cortical impact in mice. This aligns with other findings (Chahin et al., 2025), which demonstrated transient structural microglial activation 1 week post-injury in the same regions, with microglia adopting an activated morphology using a similar mouse model of injury. Moreover, observations by Drieu et al. (2022) were confirmed in this study through evidence of active phagocytosis, highlighting the functional engagement of microglia during this period. The phagocytic state was further evidenced by an increase in lysosomal volume and a shift towards hypertrophic, activated morphological phenotype, which closely matched morphological alterations described above. However, microglial synaptic pruning is tightly regulated and depends on local changes in neuronal activity and circuit excitability, enabling microglia to selectively target synapses for removal and modulate neural network function after injury. Numerous studies have demonstrated disruptions in excitatory/inhibitory balance (**Additional Table 2**), often favoring increased excitation. In this context, Chahin et al. (2025) have shown that microglia preferentially engulf excitatory synapses, likely as a compensatory mechanism to restore circuit balance. In this study, the increase in synaptic engulfment was transient following a single impact, peaking at one week and closely aligning with the timing of microglial activation. Notably, by 3 to 6 weeks post-injury, synaptic engulfment had returned to baseline levels, indicating a resolution of this microglial activity over time. However, at 3 weeks post-injury, morphological changes such as increased betweenness, greater branching complexity, and decreased sphericity remained evident, suggesting that structural remodeling of microglia may lag earlier functional changes such as synaptic engulfment. Morphological changes may also require more time to manifest because they involve cytoskeletal rearrangement and changes in cell interactions, which are slower processes than immediate phagocytic activity.

**Additional Table 2:** Summary of papers investigating neuronal and synaptic changes following mTBI and rmTBI between 2014 and 2025

Injury model	Single/repetitive mild TBI	Species	Time points post-injury	Brain regions	Synaptic changes	Neuronal loss	Structural/morphological changes	Reference
CHIMERA	Single	Mouse	7 d	Neocortex, thalamus, DG, optic tract	↓ Synaptic gene expression; ↓ synaptic proteins; ↓ synaptic density; ↓ synaptic transmission and cell-cell communication pathways	Mossy cell loss in the dentate hilus	N/A	Swaro et al., 2025
CCI	Single	Mouse	1, 3, 5, 7, 9, 10, 14, 21,42, 70 d	Sensorimotor cortex (ipsilateral region studied; contralateral side as control)	Layer II/III (21 dpi): ↑ excitability, ↑ input resistance, ↓ rheobase; layer V (5-7 dpi): ↓ excitability, depolarized resting potential, fewer spikes	Layer II/III (ATF3+ neurons): no neuronal loss Layer V (ATF3+ neurons): neuronal loss at 7 and 1 d and ↑ microglial phagocytosis	Layer II/III: axonal initial segment loss by 7 dpi → restored by 14 dpi Layer V: dendritic fragmentation and axonal beading/swelling (7 dpi)	Alkaslasi et al., 2025
Needle-induced cavitation	Single	Mouse	Acute and 3 d	Hippocampus (CA3 injury site; CA1 recordings)	Transient ↓ sEPSC frequency onto CA1 pyramidal neurons immediately post-injury ↑ in sEPSC frequency at 5-10 min post-injury	N/A	N/A	Dougan et al., 2024
WD	Single	Rat	17 d	Amygdala, frontal cortex, dorsal raphe nucleus	α1-GABA expression: ↓ in diestrus females; ↑ in males and proestrus females	N/A	N/A	Fox et al., 2023
WD	Single	Mouse	5 min, 7 d	Cerebral cortex	↓ Homerl; no change in vGlutl; ↓ vGlutl/Homerl puncta	↓ NeuN expression; ↓ PV+ interneurons; ↓ CAMKII+ neurons; no neuronal loss	Spine loss and ↑ spine head width; shift in spine types: ↑ stable mushroom spines	Munoz-Ballester et al., 2022
CFP	Single	Mouse	1 d	Cerebral cortex (somatostatin and parvalbumin interneurons)	Somatostatin interneurons: ↑ excitability; ↑ synaptic efficacy	None	N/A	Harris et al., 2022
Low-intensity blast	Single	Mouse	1 d, 1 wk, 3 mon	Hippocampus (CA3)	Acute glutamatergic hyperexcitability (↑ mEPSC frequency, bursts); chronic: ↓ synaptic plasticity proteins (3mo)	N/A	N/A	Chen et al., 2022
CCI	Single	Mouse	2 h, 12 h, 1 d	Primary motor cortex (layer II/III and V)	↓ EMG map size; ↓ Ca^2+^ amplitude; ↑ Ca^2+^ duration; ↓ AMPAR/NMDAR function; ↑ response latency	N/A	Early hypoexcitability: ↓ ipsilateral motor map size and EMG amplitude at 2 h; transient compensation: ↓ contralateral motor map size at 12h	Nguyen et al., 2021
CCI	Single	Mouse	3 wk	Right primary somatosensory cortex (S1), thalamus	Synaptic dysfunction in the thalamus: ↓ inhibitory (sIPSC) frequency; ↓ excitatory (sEPSC) amplitude; ↓ cortical input	Significant reduction in GABAergic neurons in the thalamus	N/A	Holden et al., 2021
Closed-head rotational acceleration	Single	Pig	3, 7, 30 d, 1 yr	Periventricular white matter, fimbria/fornix, hippocampus (hilus and molecular layer), corpus callosum	Synaptic pruning: ↓ spine density; ↓ synaptic transmission efficiency; ↓ LTP; changes in synaptic protein levels → synapse destabilization	None	Dendritic spines shrinking	Grovola et al., 2021
Air blast	Single	Mouse	3d	Parietal cortex (layers IV & V) and hippocampus (CA1)	↓ Synaptic connectivity → impaired neuronal communication	None	↓ Dendritic branching & distribution; ↓ dendritic length;	Ratliff et al., 2020
Closed-head rotational acceleration	Single	Pig	3, 7, 30 d, 1 yr	Hippocampus (dentate gyrus, hilus)	Acute: ↑ synapsin around mossy cells; ↓ by 30 dpi	None	N/A	Grovola et al., 2020
Low-intensity blast	Single	Mouse	7, 30 d	Cortex, hippocampus (CA1)	↓ Excitatory synapses in cortex; ↑ excitatory synapses in the hippocampus; ↓ docked vesicles in cortex; ↑ PSD-95 and synaptophysin	None	Dark neuronal perikaryon, swollen mitochondria, microtubule fragmentation, axonal vacuoles, increased tau and amyloid-beta proteins	Konan et al., 2019
Modified WD	Single	Mouse	1-48 h	Piriform and entorhinal cortices	1 h: ↑ excitatory input (sEPSC amp & freq); e/i imbalance; ↑ network reverberation 48 h: minimal changes, e/i balance stable	N/A	N/A	Witkowski et al., 2019
CFP	Single	Mouse	2d	Neocortex (primary somatosensory barrel field)	↓ Perisomatic GABAergic bouton density (layer v) → cortical hyperexcitability	N/A	↓ Axon initial segment length	Vascak et al., 2018
WD	Single	Juvenile rat	1 h, 1, 7, 28 d	Hippocampus (dentate gyrus and CA1)	Acute and persistent ↓ LTP in females; delayed, transient ↓ LTP in males	N/A	N/A	White et al., 2017
Modified WD	Single	Juvenile rat	~20 d	Cortex (prefrontal, layer III pyramidal neurons)	Impaired pruning synaptic over-connectivity	N/A	↑ Dendritic branching, ↑ dendritic length, ↑ spine density	Mychasiuk et al., 2015
CFP	Single	Rat	24-48h	Neocortex (layer V pyramidal neurons)	↑ mEPSC frequency; ↑ sEPSC frequency → network hyperexcitability	N/A	N/A	Hanelletal., 2015
CCI	Single	Rat	1-7 d	Hippocampal CA1 region	↓ LTP; ↓ NMDA receptor currents; ↓ GABA_a_ receptor-mediated IPSCs; ↓ surface expression of GABA_a_ receptor subunits	Selective loss of GABAergic interneurons	N/A	Almeida-Suhett et al., 2015
CCI	Single	Rat	1,7, 30 d	Basolateral amygdala	↓ GABA_a_ receptor-mediated IPSC; ↓ surface expression of GABA_A_ receptor subunits ↑ α7-nAChR currents and surface expression	Selective loss of GABAergic interneurons	N/A	Almeida-Suhett et al., 2014
CHI with CVC	Repetitive (x14)	Mouse	6 wk	Olfactory areas, thalamus, midbrain	↓ Synaptic density; ↑ BOLD signal homogeneity	None	N/A	Markicevic et al., 2024
CHIMERA	Repetitive (x3)	Rat	4 d	Optic tract, lateral geniculate nucleus	None	N/A	White-matter axon degeneration	Boese et al., 2024
CCI	Repetitive (x3)	Mouse	1 wk	Cortex, hippocampus	↓ Genes linked to synaptic plasticity, neuronal communication, and neurotransmitter regulation; ↑ senescence markers	N/A	N/A	Schwab et al., 2022
CCI	Repetitive (x3)	Mouse	2 wk	Hippocampus	Persistent postsynaptic modifications in AMPAR and GABA_a_ receptor function; e/i balance shifted toward inhibition (↓ e/i ratio)	N/A	Altered receptor subunits (↓ GluR1/GluR2, ↓ GABA_a_ subunits α1, β2/3, γ2	Langlois et al., 2022
Lateral impact model	Repetitive (x5)	Mouse	1-5 d	Motor cortex (layer III pyramidal neurons); granular insular cortex (prefrontal cortex)	↓ Spine density in prefrontal cortex → excessive synaptic pruning ↑ spine density in motor cortex → impaired synaptic pruning	N/A	N/A	Eyolfson et al., 2022
High-frequency head impact	Repetitive (x30)	Mouse	1 d	Hippocampus	↓ Early-LTP in CA1; ↑ Ca^2+^ transient frequency,	None	N/A	Chapman et al., 2022
CCI	Repetitive (x5)	Mouse	8-48 h; 2, 6, 10, 16 wk	Prefrontal cortex, hippocampus, corpus callosum	↓ Synaptophysin levels (acute and chronic); ↑ excitatory marker PSD-95; ↓ inhibitory marker gephyrin acute → e/i imbalance	N/A	Reduced myelin thickness, increased demyelinated axons, and myelin decompaction	Xu et al., 2021
High-frequency head impact	Repetitive (x30)	Mouse	1 d, 1 mon	Hippocampus (CA1, CA3)	Dysregulation of synaptic genes; ↓ LTP & AMPA/NMDA ratio; ↓ excitability; ↓ mEPSC	No neuronal loss	N/A	Sloley et al., 2021
CHIMERA	Repetitive (x6)	Rat	1, 7, 28 d, 2-3 mon	Medial prefrontal cortex	↑ mEPSC amplitude; ↓ mIPSC frequency; ↓ presynaptic GABA release regulation (↓ inhibition)	No	N/A	Feng et al., 2021
Air blast	Repetitive (x3)	Mouse	2, 7 d, >30 d	Dentate gyrus, CA1	↑ EPSC amplitude; ↓ latency; ↓ threshold	N/A	N/A	Bugay et al., 2020
CCI	Single/repetitive (x3)	Mouse	48 h, 1, 3, 6 wk	Sensorimotor cortex (layer II/III) and hippocampus (CA1, CA3, dentate gyrus)	Single → transient ↑ vGlut^+^ engulfment; vGat+ engulfment unchanged Repetitive → sustained ↑ vGlut^+^ engulfment; vGat^+^ engulfment unchanged	None	No change in total spine density (layer II/III cortex) Single: no change in spine morphology Repetitive: ↑ in immature, stubby spines	Chahin et al., 2025
CFP	Single/repetitive (x2)	Mouse	1 d	Neocortex (layer V)	N/A	None	c-Jun+ neurons: ↑ cell and nuclear volume → hypertrophy; NeuN-neurons: ↓ cell and nuclear volume → atrophy	Ogino et al., 2022
Air blast	Single/repetiti ve (x4)	Mouse	4, 15 mon	Hippocampus	N/A	Repetitive: dorsal hippocampal neuron loss	N/A	Honig et al., 2021
CCI	Single/repetitive	Rat	30 d	Hippocampus (CA1, cortex)	Single/repetitive: ↑ synaptic excitability; ↑ resting and voltage-gated Ca^+^ signals;	None	Single and repetitive: ↑ phospho-tau	McDaid et al., 2021
LFP	Single/repetitive (x3)	Rat	28 d	Hippocampus (bilateral CA1, CA3, dentate gyrus)	Single: ↑ AMPA/NMDA ratio Repetitive: → ↓ NMDA-mediated responses; AMPA responses unchanged; ↑↑ AMPA/NMDA ratio	Single → neuronal loss in contralateral cortex and hippocampus (bilateral) Repetitive → significant neuronal loss cortex and hippocampus (bilateral).	N/A	Aungst et al., 2014

ATF3: Activating transcription factor 3; amp: amplitude; AMPA: α-amino-3-hydroxy-5-methyl-4-isoxazolepropionic acid; CA1, CA3: cornu ammonis regions 1 and 3 of the hippocampus; CCI: controlled cortical impact; CFP: central fluid percussion; CHI: closed-head injury; CHIMERA: closed-head impact model of engineered rotational acceleration; CVC: chronic variable stress; DG: dentate gyrus; d: day(s); dpi: days post-injury; e/i: excitatory/inhibitory; EMG: electromyogram; freq: frequency; GABA: gamma-aminobutyric acid; Glu: glutamate; h: hour(s); LTP: long-term potentiation; min: minutes; mEPSC: miniature excitatory postsynaptic current; mon: month(s); mTBI: mild traumatic brain injury; rmTBI: repetitive mild traumatic brain injury; N/A: not available; nAChR: nicotinic acetylcholine receptor; NeuN: neuronal nuclear protein; NMDA: N-methyl-D-aspartate; PSD-95: postsynaptic density protein 95; PV: parvalbumin; R: receptor; sEPSC: spontaneous excitatory postsynaptic current; sIPSC: spontaneous inhibitory postsynaptic current; vGat: vesicular GABA transporter; vGlut: vesicular glutamate transporter; wk: week(s); WD: weight drop; yr: year(s); c-Jun: protein of the activator protein-1 complex; ↑: increase/upregulation; ↑↑: strong increase/marked upregulation; ↓: decrease/downregulation; →: transformation or transition.

In the same mild TBI model in mice, Taib et al. (2017) focused on the cortex and observed dynamic changes in immunoregulatory marker levels during the first week post-injury. More specifically, pro-inflammatory signals peaked early between 6 and 24 hours post-injury, followed by a transient anti-inflammatory peak around days 2 to 3, with most markers returning to baseline by day 7. This complex pattern suggests that microglia dynamically regulate their activation state after mild injury, balancing inflammatory and reparative processes during the critical early recovery period. Building on this, other studies have also reported an upregulation of inflammatory and phagocytic genes around 1 week post-injury, coinciding with the timing of microglial activation observed in our study (Chahin et al., 2025). These molecular changes, detected in different brain regions, align with increased microglial engagement in tissue remodeling and clearance during the subacute phase using models of mild TBI such as the closed-head impact model of engineered rotational acceleration or the controlled cortical impact in mice (Alkaslasi et al., 2025; Swaro et al., 2025). This convergence underscores a coordinated temporal window during which microglial cells actively contribute to neuroinflammatory and reparative processes following mild TBI.

Although microglial activation after a single mild TBI is generally transient, Grovola et al. (2021) observed persistent microglial reactivity lasting up to 1 year post-injury in the rapid head rotation pig model. This prolonged activation may be attributed to the diffuse shear forces induced by rotational acceleration injuries, which affect widespread white matter tracts and multiple brain regions. Such extensive mechanical stress likely drives a sustained neuroinflammatory environment, contrasting with the more localized and transient microglial responses typically seen in other mild TBI models. Another recent study using a weight-drop model in mice examined varying severities within mild TBI and attributed different levels of microglial activation to injury burden. They found that more severe mild injuries involving intracerebral bleeding (degrees III and IV) triggered pronounced microglial proliferation and morphological changes across all cortical layers, with a peak around 3 days post-injury (Nespoli et al., 2024). These findings highlight that both the nature (rotational versus impact) and severity (presence of bleeding) of mild TBI critically determine the spatial extent and temporal persistence of microglial activation. This underscores the importance of considering injury biomechanics and pathology when interpreting microglial responses and designing targeted interventions for mild TBI. Finally, it should be mentioned that the diversity of animal models and the wide range of injury paradigms pose major challenges in drawing consistent conclusions between studies.

Building upon the understanding of microglial dynamics following single mTBI, numerous studies have investigated the effects of repetitive injuries, revealing distinct and often more pronounced neuroimmune responses. Repetitive mTBI models, which better mimic clinical scenarios such as sports-related concussions, consistently show amplified responses compared to single insults.

**Figure 4:**
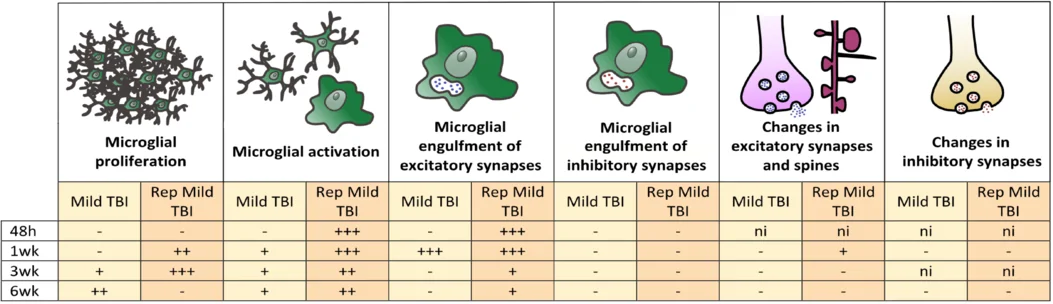
Schematic of the changes triggered by mild TBI and repetitive mild TBI. These alterations in a controlled cortical impact model in mice regarding microglia proliferation, microglia activation, microglia engulfment of synaptic material, and synapse density were based on Chahin et al. (2025). Created with BioRender.com. -: Not observed; +: mild occurrence; ++: moderate occurrence; +++: strong occurrence; ni: not investigated; Rep: repetitive; TBI: traumatic brain injury.

Compared to single mTBI, where microglial activation typically peaks around 1 week and diminishes within 3 to 6 weeks, Chahin et al. (2025), using a closed-head cortical impact in mice with three repetitions, demonstrated that repetitive mild TBI induces a significantly prolonged microglial response. Sustained microglial activation was observed, reflected by an increase in engulfment activity, exclusively of excitatory synaptic material, lasting at least 6 weeks post-injury (**Figure 4**). This clearly demonstrates extended neuroimmune engagement following repeated concussions compared to the more transient activation seen after a single mild TBI.

Adding to these findings, Schwab et al. (2022) used transcriptomic profiling to reveal a persistent upregulation of inflammatory and phagocytic genes in microglia after three repetitive closed-skull controlled cortical impacts in mice. Their data highlight a chronic neuroinflammatory state characterized by metabolic dysregulation and a senescence-associated secretory phenotype, suggesting that microglial dysfunction may contribute to long-term brain pathology. Interestingly, persistent microglia activation is not seen only at the site of injury or in close areas to the mild injury but can be detected in anatomically remote brain regions, including the white matter. This activation, which can be found up to 1 year post-injury, is considered a key player in the chronic neurodegenerative consequences of TBI (Shitaka et al., 2011; Bolton Hall et al., 2016; Brooks et al., 2017; Mouzon et al., 2019; Velayudhan et al., 2021). Interestingly, Raghupathi et al. (2023) demonstrated in neonatal rats that repetitive, but not single, mild closed-head CCI injury led to sustained cognitive deficits accompanied by chronic microglial activation and neurodegeneration in white matter and thalamus. Similarly, in a large animal model, Grovola et al. (2023) found that rotational mTBI in pigs produced transient axonal pathology in the periventricular white matter accompanied by microglial morphological changes that resembled activation, paralleling rodent findings and underscoring the translational relevance of persistent glial responses. Clinically, mild TBI has also been linked to enduring white matter damage: in a prospective cohort, Hellstrøm et al. (2017) reported diffusion tensor imaging abnormalities at 12 months post-injury that correlated with persistent clinical symptoms, particularly in callosal tracts. Yet, the functional role of activated microglia in the white matter remains complex. While animal data tend to emphasize their contribution to chronic white matter injury, recent clinical work suggests their role may shift over time. In a longitudinal study of mTBI patients, Jia et al. (2025) showed that microglial gene expression signatures were associated with reduced white matter atrophy at 12 months, in contrast to endothelial and excitatory neuron markers that predicted worse outcomes. This raises the possibility that microglial responses may be initially protective, perhaps limiting secondary degeneration, but in other contexts or with repetitive injury, they may exacerbate damage. Taken together, these findings point to a dual and stage-dependent role for microglia in white matter after mTBI.

Numerous other studies have similarly reported sustained microglial activation following repetitive mild TBI, often employing three or more impacts. This persistent response is widespread across multiple brain regions and, in some cases, shows clear sex-specific pathology (Robinson et al., 2017; Neale et al., 2023). Across these studies, there is a consistent description of microglial morphological shifts toward hypertrophic, swollen, and bushy phenotypes, hallmarks of heightened reactivity and phagocytic function (Shitaka et al., 2011; Tyburski et al., 2017; Chahin et al., 2025; Kaiser et al., 2025; Sugahara et al., 2025). Collectively, these converging findings underscore the widespread and prolonged nature of microglial activation after repetitive injury (**Additional Table 1**), independent of injury type and animal models.

*In vivo* imaging studies using TSPO PET are widely used to demonstrate microglial activation after TBI in both animal models and humans. In mTBI, TSPO PET imaging studies have shown variable results compared to moderate or severe TBI. Some animal studies have found increased TSPO expression suggestive of microglial activation in brain regions shortly after mild injury, but PET signal changes were often subtle or absent depending on the ligand and timing (Donat et al., 2017; Delage et al., 2021). Flow cytometry and immunohistochemistry have revealed that TSPO expression after mTBI includes not only microglia but also other immune and brain cells, limiting the specificity of TSPO as a pure marker of microglial activation. In people with mild or repetitive brain injury, PET with second-generation TSPO ligands has shown increased microglial activation, particularly in regions of white matter damage and in the medial temporal lobes (De Picker et al., 2023; Giarratana, 2025). However, the extent of TSPO upregulation after mild injury tends to be lower and more localized than in moderate to severe cases. Overall, TSPO PET can detect neuroinflammation in mTBI, but due to its limited sensitivity and specificity, it may underestimate the full spectrum and complexity of microglial and immunological responses in mild brain injury.

### Microglial activation pathways and receptor signaling in mild traumatic brain injury

mTBI triggers microglial activation via signals from damaged neurons, astrocytes, and endothelial cells. Neuronal release of damage-associated molecular patterns (e.g., ATP, HMGB1, glutamate) activates nearby microglia, while astrocytes and BBB endothelial cells release cytokines (e.g., IL-1β and TNF-α) and adhesion molecules (intercellular adhesion molecule 1 and vascular cell adhesion molecule 1), which further amplify inflammation. If the BBB is compromised, peripheral immune cells infiltrate the brain, escalating the inflammatory cascade (Liu et al., 2023; Mayer et al., 2024; Zhang et al., 2025).

Their activation state ranges from pro-inflammatory to anti-inflammatory, with pro-inflammatory microglia promoting cytokine and reactive oxygen species release, and anti-inflammatory microglia supporting tissue repair via factors such as IL-10, Arg1, and insulin-like growth factor 1 (Guo et al., 2022). As nicely summarized by Shao et al. (2022), various molecular regulators of microglial/macrophage polarization, including NOX2, HDACs, HMGB1, and TGF-β1, can be targeted to suppress pro-inflammatory microglia activation while promoting the anti-inflammatory phenotype (Wang et al., 2015, 2017; Gao et al., 2018; Zhao et al., 2020). Targeting such regulators can modulate inflammation, protect neurons, and improve recovery after TBI. For example, the C3aR antagonist SB 290157 reduced inflammatory microglial migration and phagocytic activity in a mouse model (Surugiu et al., 2019). However, these findings are largely derived from moderate TBI models, while research specifically addressing microglial polarization and therapeutic regulation in mild TBI remains limited.

Simultaneously, growing evidence suggests that the binary pro-inflammatory/anti-inflammatory framework itself may be too simplistic to capture the full spectrum of microglial phenotypes *in vivo*. The traditional polarization scheme, derived from *in vitro* cytokine stimulation, does not adequately reflect the complex and dynamic environments microglia experience in the injured brain. Genome-wide and single-cell transcriptomic studies have shown that microglia often co-express markers from both so-called polarized states and instead display diverse, context-dependent responses shaped by their CNS environment (Ransohoff, 2016; Jassam et al., 2017), existing as a continuum between resting and activation states. Consistent with this, high-throughput transcriptomic analyses, including single-cell RNA sequencing, have identified transcriptional profiles that co-express markers associated with both pro- and anti-inflammatory phenotypes, suggesting a broader and more dynamic spectrum of microglial states shaped by injury context and cellular interactions (Donat et al., 2017; Masuda et al., 2020). These studies highlight microglial activation as a dynamic and heterogeneous process rather than a binary switch, pointing toward the need to reinterpret microglial responses in TBI within this broader framework.

Across this expanded spectrum, distinct microglial subpopulations have been described. Some emerge in response to inflammation or neurodegeneration, such as the inflammation-associated phenotype (Sousa et al., 2018) and the disease‐associated phenotype state first described in ALS models (Keren-Shaul et al., 2017). Others are tied to developmental programs: axon tract–associated microglia localize to white matter and contribute to axon remodeling and myelination (Hammond et al., 2019), while proliferative region–associated microglia arise in postnatal neurogenic niches to support brain maturation (Li et al., 2019). Beyond development and degeneration, microglia can also adopt a transient recovery-associated state during the resolution of injury, highlighting their role in restoring CNS homeostasis (Tay et al., 2018). Collectively, accumulating evidence demonstrates that microglial identity is highly dynamic, varying with developmental stage, spatial context, and disease environment (Masuda et al., 2019).

Although these studies have expanded our understanding of microglial heterogeneity, most have focused on neurodegenerative disease or developmental programs, leaving comparatively less known about how microglial states are shaped by TBI. However, in recent years, important insights have begun to emerge. For example, single-cell RNA sequencing of FACS-isolated microglia has revealed distinct transcriptional clusters associated with host defense, synaptic plasticity, lipid remodeling, and membrane polarization over the long-term course of injury (Makinde et al., 2020). These longitudinal adaptations suggest that microglia not only contribute to acute injury responses but may also underlie the persistence of neurological impairments, emphasizing their central role in both neuropathogenic and neurorestorative processes. However, such work has largely relied on models of moderate-to-severe TBI, but complementary insights have also emerged from studies of mild TBI. In a hippocampal Drop-seq analysis performed 24 hours after mild concussive FPI, Arneson et al. (2018) identified more than 50 differentially expressed genes in microglia, many linked to inflammatory and immune pathways. These findings underscore the rapid responsiveness of microglia to even mild injury and highlight their potential role in initiating neuroinflammatory cascades that may shape long-term outcomes (Arneson et al., 2018). A subsequent reanalysis of the same dataset (GSE101901) expanded on these observations, revealing that hippocampal microglia not only differentially express immune-related genes but also activate a broader inflammatory program. Pathways such as IL-18 signaling, chemokine–receptor interactions, NOD-like receptor signaling, and TYROBP networks were upregulated, pointing to a multifaceted pro-inflammatory response. Interestingly, unlike cortical microglia, which preferentially engage interferon signaling after TBI, hippocampal microglia did not show strong interferon pathway activation, underscoring regional heterogeneity in their transcriptional states (Xing et al., 2022). Another particularly insightful study was the direct comparison between severe TBI transcriptomes and those previously reported following mild TBI by Arneson et al. (2018). In microglia, there was substantial overlap between the two models, with shared changes dominated by interferon-stimulated genes (*Ifit1*, *Ifit3*, *Ifi27,* *Oasl2*, and *Stat2*), damage-associated genes (*Lgals3bp*, *Lpl*, and *Clec7a*), and multiple ribosomal subunits. However, the severe TBI model revealed a far broader immune activation, with unique enrichment for type I interferon signaling and MHC class I antigen presentation pathways, whereas the mild TBI microglial response was more restricted and dominated by genes involved in general cellular processes rather than overt immune defense (Todd et al., 2021).

While these studies focused on parenchymal microglia, recent work has begun to extend this analysis to other compartments and brain regions, revealing that glial responses to TBI are highly context-dependent. For example, Bolte et al. (2023) applied single-cell and bulk RNA sequencing to the meninges after mild closed-skull TBI in mice and reported an expansion of meningeal macrophages that upregulated type I interferon–associated genes, highlighting that border-associated myeloid cells mount an immune response distinct from that of hippocampal microglia. Similarly, Zhuang et al. (2025) combined single-nucleus RNA sequencing and spatial transcriptomics to generate a cellular atlas of the mouse brainstem under normal conditions and after weight drop mTBI. They identified microglia as one of the major non-neuronal populations; however, unlike in the hippocampus, brainstem microglia showed only minimal changes in abundance within the first 24 hours post-injury, suggesting a relatively restrained inflammatory response in this region. By contrast, other glial and neuronal populations exhibited more pronounced alterations (Zhuang et al., 2025). Together, these findings emphasize that glial responses to TBI vary not only with injury severity but also across anatomical compartments and brain regions.

Complementing these insights, Swaro et al. (2025) employed spatial transcriptomics in the closed-head impact model of engineered rotational acceleration to examine glial dysregulation across multiple brain regions. Their analysis revealed that microglial dysregulation was not uniformly distributed but instead varied markedly across regions. In white matter tracts such as the optic nerve, microglia upregulated genes linked to reactivity, including *CD68*, *CTSD*, and *APOE*, consistent with a phagocytic and inflammatory phenotype. By contrast, brainstem and cortical microglia showed far fewer changes at this subacute stage, suggesting that their activation is more restrained compared to hippocampal or thalamic regions. These observations underscore that even after mild diffuse TBI, microglial responses are highly spatially heterogeneous, with certain compartments mounting strong inflammatory programs while others remain relatively unchanged.

In addition to these region- and context-specific differences, microglial activation after mTBI is also governed by receptor-mediated signaling that shapes the inflammatory response. Toll-like receptor 4, a pattern recognition receptor expressed on microglia, plays a dual role in rmTBI depending on the timing of activation. Early Toll-like receptor 4 signaling can limit inflammation and neuronal damage, while delayed activation exacerbates cytokine release, tau pathology, and cognitive decline (Corrigan et al., 2017). Similarly, the nuclear receptor PPARγ has emerged as a key regulator of microglial inflammation. Pearson et al. (2024) showed that pharmacological activation of PPARγ with pioglitazone reduced chronic neuroinflammation and improved cognitive outcomes post-injury. Furthermore, microglial-specific overexpression of PPARγ suppressed pro-inflammatory signaling pathways and prevented a disease-associated phenotype.

Similarly, Bu et al. (2016) showed that targeting cannabinoid type 2 (CB2) receptors with the inverse agonist SMM-189 modulates microglial phenotype after blast-induced mTBI in mice. SMM-189 increased nuclear pCREB in microglia, promoting a shift from pro-inflammatory to reparative states. This shift was associated with partial rescue of cortical, striatal, and amygdalar neurons, particularly fear-suppressing populations in the basolateral amygdala. The intervention significantly reduced motor deficits and fear-related behaviors, underscoring CB2 inverse agonists as a potential strategy for mitigating neuroinflammation and preserving neuronal integrity post-TBI. The therapeutic potential of SMM-189 has been supported by other studies as well, such as Reiner et al. (2015), which highlighted its role in reducing neurobehavioral impairments and visual system pathology following mild TBI through modulation of CB2 receptor activity.

Despite extensive characterization of microglial receptor pathways in moderate and severe TBI, as well as in other neuroinflammatory conditions, our understanding of receptor-specific microglial dynamics in mTBI remains limited. While receptors such as Toll-like receptor 4, PPARγ, and CB2 have shown promise in modulating outcomes post-injury, most data are preliminary and focus on acute stages. There is a clear need for more comprehensive, receptor-focused studies in mTBI to clarify how microglia contribute to long-term outcomes and to identify viable targets for early therapeutic intervention.

### Microglial metabolic reprogramming

Metabolic reprogramming of microglia is increasingly recognized as a central mechanism in shaping the inflammatory and reparative responses following TBI. Recent studies have shown that microglia are highly adaptable and alter their metabolism in response to injury and environmental cues in the injured brain. These metabolic changes, particularly changes in mitochondrial function and glycolytic activity, regulate the polarization of microglia towards a pro-inflammatory or anti-inflammatory state, which has direct implications for neurodegeneration and tissue repair following traumatic brain injury (Strogulski et al., 2023). After TBI, microglia rapidly shift from oxidative phosphorylation to increased glycolysis, a shift that favors the production of inflammatory cytokines and exacerbates secondary injury and neuroinflammation. Disrupted mitochondrial metabolism impairs energy supply and increases pro-inflammatory activation, contributing to chronic neuronal damage. However, the intake of specific metabolites such as itaconate can partially restore the healthy bioenergetics of microglia, reduce inflammation, and promote neurological recovery (Liu et al., 2025). Conversely, the promotion of oxidative metabolism in microglia supports their anti-inflammatory phenotype and reparative functions. Certain metabolic pathways, such as glutaminolysis and fatty acid oxidation, are associated with tissue repair and improved outcomes following injury. These processes are essential for the resolution of inflammation, phagocytosis of cellular debris, and restoration of neural tissue integrity.

In summary, metabolic reprogramming of microglia after traumatic brain injury is a central axis that determines the balance between neuroinflammation and repair, and offers potential therapeutic targets to improve outcomes following brain injury.

## Synaptic Changes and Changes in Excitatory/Inhibitory Brain Balance

mTBI, despite its classification as clinically minor, can lead to profound and persistent changes in brain function. A growing body of research indicates that these injuries can alter neuronal excitability, synaptic structure, and the balance between excitatory and inhibitory signaling in key brain regions, often without overt structural damage detectable via conventional imaging. The following paragraphs synthesize findings from multiple studies, highlighting the converging evidence for functional and synaptic disruptions in mTBI (**Additional Table 2**).

One of the earliest and most consistent effects of mTBI appears to be a disruption in the balance between excitatory and inhibitory synaptic activity. Witkowski et al. (2019) demonstrated that a single mild TBI causes a swift increase in excitatory synaptic input within the piriform and entorhinal cortices as early as 1 hour post-injury, leading to transient hyperexcitability without major changes in inhibitory transmission or synapse number. This early imbalance was associated with behavioral deficits in olfactory discrimination, highlighting the immediate functional impact of mild injury. This synaptic imbalance often manifests with region-specific dynamics. For example, Konan et al. (2019) reported decreased synapse density in the cortex alongside increased synapses in the hippocampus following mild blast injury, suggesting complex spatial remodeling rather than uniform synaptic loss. Complementing these findings at the functional level, Hånell et al. (2015) demonstrated that a single mild diffuse brain injury induces increased excitatory synaptic input within neocortical networks during the acute post-injury period. Specifically, axotomized neurons exhibit an early increase in excitatory synaptic input, detectable as soon as 24 hours post-injury (Hånell et al., 2015). In contrast, structurally intact neurons show a similar increase, but with a delayed onset around 48 hours. Despite the heightened input, axotomized neurons display reduced intrinsic excitability and fire less frequently, likely due to impaired signal conduction caused by axonal damage (Greer et al., 2012). Conversely, intact neurons exhibit increased intrinsic excitability and elevated firing rates, though this heightened activity emerges later in the post-injury period. However, these intact neurons also lose their intrinsic bursting pattern, a disruption that may impair neural coding and synchronization. This divergence in neuronal behavior reflects a disruption in network coordination, where diminished output from damaged neurons is not effectively compensated by the delayed hyperactive firing from intact neurons, potentially contributing to broader circuit instability and functional deficits (Greer et al., 2012; Hånell et al., 2015). Similarly, Chen et al. (2022) found that mild blast injury induces acute glutamatergic hyperexcitability in hippocampal CA3 neurons, dependent on NMDA receptor activation. This hyperexcitability is normalized by 3 months post-injury. However, synaptic plasticity proteins remain reduced, correlating with persistent cognitive deficits in learning and cognitive flexibility (Chen et al., 2022). While these studies underscore region-specific alterations in excitability and synaptic structure, recent findings reveal that such changes can also occur within the same brain region in a layer-specific manner. In the sensorimotor cortex, for example, superficial layer II/III neurons become more excitable following mild TBI, requiring less stimulation to fire and showing no significant neuronal loss. In contrast, deeper layer V neurons respond with decreased excitability, more depolarized resting membrane potentials, and progressive structural degeneration, including dendritic and axonal damage. Interestingly, layer II/III neurons also undergo a transient loss of the axon initial segment around 1 week post-injury, which is restored by the second week, suggesting a potential adaptive recovery mechanism (Alkaslasi et al., 2025). These divergent responses underscore the complexity of neuronal remodeling and differential vulnerability within cortical layers following mild brain injury.

While these findings highlight critical alterations in excitatory synaptic input and neuronal firing, effective brain function relies equally on inhibitory signaling to maintain network balance. Disruptions in inhibitory circuits, such as those involving GABAergic interneurons and GABA_A_ receptors, can exacerbate network instability and contribute to the cognitive and behavioral deficits observed after mTBI. In the hippocampal CA1 region, a selective loss of GABAergic interneurons was accompanied by downregulation of key GABA_A_ receptor subunits (α1, β2/3, and γ2), leading to reduced inhibitory signaling, as reflected by diminished frequency and amplitude of GABA_A_ receptor–mediated IPSCs (Almeida-Suhett et al., 2015). Concurrently, N-methyl-D-aspartate (NMDA) receptor–mediated excitatory currents were diminished, whereas AMPA receptor–mediated transmission remained unchanged. These synaptic alterations led to impaired long-term potentiation (LTP), a key mechanism underlying learning and memory. Supporting this, White et al. (2017) observed LTP deficits in both the dentate gyrus and CA1 regions of the hippocampus in juvenile rats following a single mTBI, with impairments emerging earlier and lasting longer in females. These findings emphasize the vulnerability of hippocampal circuits to long-lasting synaptic disruption following even mild brain injury. However, similar patterns of inhibitory dysfunction are also evident in the basolateral amygdala, where the loss of GABAergic interneurons and reduced surface expression of GABA_A_ receptors occur alongside increased activity of α7-nicotinic acetylcholine receptors (Fox et al., 2023). These changes shift the local circuit toward a hyperexcitable state, potentially impairing amygdala-dependent functions, resulting in heightened anxiety-like behavior observed after mTBI (Almeida-Suhett et al., 2014). Extending the pattern of disrupted GABAergic signaling beyond previously described regions, Holden et al. (2021) reported that mild TBI causes secondary damage to the thalamus, particularly the nucleus reticularis thalami, which is marked by a significant loss of GABAergic neurons. This disruption leads to decreased inhibitory synaptic input and reduced excitatory drive to the nucleus reticularis thalami, impairing thalamic inhibition and destabilizing cortical rhythms such as sleep spindles, oscillations critical for sensory processing and memory consolidation. While previous studies have broadly demonstrated the loss or downregulation of GABAergic signaling following mTBI (Vascak et al., 2018), more recent work has begun to reveal heterogeneity within inhibitory circuits. Expanding on this, Harris et al. (2022) identified subtype-specific responses among cortical interneurons, showing that parvalbumin (PV) interneurons, key regulators of fast, synchronous inhibition, undergo axotomy shortly after injury. In contrast, somatostatin interneurons, which provide dendritic inhibition, were spared and instead became hyperexcitable. This increased somatostatin activity provided compensatory inhibitory input onto pyramidal neurons, potentially helping to stabilize excitatory/inhibitory balance in the context of PV interneuron loss. Supporting this, Muñoz-Ballester et al. (2022) reported reduced densities of PV^+^ interneurons and CaMKII^+^ excitatory neurons, along with decreased expression of Homer1, a postsynaptic scaffolding protein involved in excitatory transmission. These molecular and cellular changes further underscore the widespread disruption of synaptic organization following mTBI.

These changes in synaptic signaling may increase synaptic vulnerability to pruning, disrupting structural plasticity and circuit refinement. Chahin et al. (2025) provided detailed evidence showing that even in the absence of overt neuronal loss or gross tissue damage, a mild concussion can initiate active synaptic remodeling through microglia-synapse interactions. Using a bilateral controlled cortical impact model in mice, they observed a clear but temporary increase in microglia-mediated vGlut^+^ presynaptic excitatory synaptic pruning in both the cortex and hippocampus shortly after a single injury. Even though the overall number of excitatory synapses remained stable over six weeks, this sudden increase in microglial activity suggests a period of active microglia-mediated synaptic remodeling rather than simple degeneration. Interestingly, microglia-mediated vGat^+^ inhibitory synapses were unaffected, pointing to a selective vulnerability of excitatory connections. These findings reveal a subtle yet meaningful way in which mild TBI may reshape brain circuits, modifying excitatory input without causing obvious damage, and potentially contributing to long-lasting cognitive difficulties (**Figure 4**). This is reminiscent of studies on moderate TBI, which emphasize synaptic remodeling (Empl et al., 2025) and the strong link of this remodeling to microglia-mediated engulfment (Krukowski et al., 2021; Wang et al., 2023). These studies show that microglia directly engulf synaptic elements, contributing to the synaptic loss associated with cognitive impairment, in particular learning and memory. Building on these findings, Grovola et al. (2020), using a porcine model of mild TBI, reported a transient increase in synapsin staining around mossy cells in the dentate gyrus hilus shortly after injury, which normalized by 30 days post-injury, further supporting the idea of dynamic synaptic remodeling rather than permanent loss. These structural changes were associated with reduced synaptic transmission efficiency and impaired long-term potentiation, indicating synaptic destabilization. In a related study using the same porcine model, Grovola et al. (2021) extended these observations by identifying synaptic pruning deficits, reduced spine density, and decreased levels of synaptic proteins such as postsynaptic density protein 95 (PSD-95) and synaptophysin across memory-related brain regions. These alterations were linked to impaired synaptic function and reduced plasticity. Supporting a molecular basis for these structural deficits, Mychasiuk et al. (2015) found that a single mTBI in juvenile rats impairs synaptic pruning and leads to excessive connectivity in the medial prefrontal cortex. This was accompanied by reduced expression of plasticity-related genes such as *Dnmt1*, *Bdnf*, and *Igf1*, alongside increased expression of growth-inhibitory factors like *Nogo-A* and *Fgf2*, suggesting that mTBI disrupts the molecular regulation of synaptic refinement. Most recent evidence further elucidates the molecular mechanisms of synaptic disruption following mild TBI, as demonstrated by Swaro et al. (2025), who used spatial transcriptomics to map brain-wide gene expression changes 1 week after a single mild TBI. They observed widespread downregulation of key synaptic communication genes such as *Syngr1*, *Adgrb1*, and *Nrxn2* in cortical regions, along with reduced expression of the excitatory synapse regulator *Nptxr*. In the thalamus, there was a region-specific decrease in genes involved in glutamatergic transmission, including the NMDA receptor subunit *Grin2c* and AMPA receptor auxiliary subunits *Shisa6* and *Shisa9*.

These structural and functional alterations reflect the attempt of the brain to adapt and reorganize synaptic connections in response to injury. Dendritic spines, which are the primary sites of excitatory synaptic input, are particularly vulnerable to damage following mild TBI. Changes in spine morphology, such as shifts from thin, plastic spines to more stable mushroom-shaped spines, can signify a disruption in synaptic plasticity and connectivity. A recent study demonstrated that no changes were observed after a single mTBI (Chahin et al., 2025). Spine density and the relative proportions of spine types in layer II/III cortical neurons remained largely unchanged. However, other studies suggest that morphological changes to dendrites and spines can occur following single hits, potentially depending on factors such as injury model, brain region, or post-injury timing. For example, Muñoz-Ballester et al. (2022) observed significant spine remodeling characterized by increased stable mushroom spines and decreased thin, plastic spines, indicative of altered synaptic connectivity. Likewise, Ratliff et al. (2020) reported reduced dendritic branching, length, and complexity in the parietal cortex and hippocampus shortly after blast injury, changes that correlated with cognitive and behavioral deficits despite the absence of neuronal loss. These structural modifications, especially at dendritic spines, underline the morphological adaptations for synaptic dysfunction following mild brain injury.

Having outlined the synaptic and neuronal changes following a single mild TBI, it is crucial to recognize that such an initial injury increases the brain’s vulnerability to subsequent concussions within a critical time window (Laurer et al., 2001), thereby amplifying synaptic dysfunction and neuronal remodeling with repetitive impacts. The following section explores these cumulative effects in detail in the case of rmTBI.

Excitatory/inhibitory imbalance persists and often worsens following repetitive mild TBIs, amplifying network dysfunction and increasing seizure susceptibility. Bugay et al. (2020) demonstrated that three repeated blast exposures in mice led to increased amplitude of excitatory postsynaptic currents and heightened intrinsic excitability in the dentate gyrus and CA1 regions. These synaptic changes were associated with non-convulsive seizures progressing to chronic epilepsy. Complementing these findings, Honig et al. (2021) used a four-exposure repetitive air blast model in mice and observed progressive hippocampal neuron loss alongside microglial dysfunction and vascular abnormalities, which correlated with long-term impairments in spatial and contextual memory tasks. Extending these hippocampal synaptic changes, Langlois et al. (2022) observed enhanced postsynaptic excitation, reduced presynaptic glutamate release, and increased inhibitory input with faster GABAergic decay in the hippocampal CA1 region. These changes shifted the excitation/inhibition balance toward inhibition and were accompanied by decreased expression of AMPA receptor subunits (GluR1/GluR2) and GABA_A_ receptor subunits (α1, β2/3, and γ2). Behaviorally, these synaptic disruptions correlated with impaired working and spatial memory, indicating a persistent synaptic imbalance contributing to cognitive deficits. McDaid et al. (2021) extended these findings by showing that both single and repetitive mild closed-head injuries in rats lead to persistent hippocampal synaptic hyperexcitability and calcium dyshomeostasis. Increased resting intracellular calcium and voltage-gated calcium channel activity were observed after injury. Notably, repetitive injury specifically caused a reduction in ryanodine receptor-evoked calcium release and widespread phospho-tau accumulation, revealing exacerbated postsynaptic dysfunction and neurodegenerative pathology beyond that seen with a single injury. Further studies in the medial prefrontal cortex demonstrated that six repetitive mild impacts in rats induce a persistent excitation/inhibition imbalance, characterized by increased excitatory synaptic strength and reduced inhibitory input due to mitochondrial dysfunction impairing presynaptic GABA release. Despite intact glutamatergic release and absence of overt motor deficits, these synaptic alterations were associated with long-term cognitive impairments, including deficits in fear memory and recognition memory, underscoring the complex and region-specific consequences of repetitive mild TBI (Feng et al. 2021). Schwab et al. (2022) further linked synaptic dysfunction to cognitive and anxiety-like behavioral deficits following repetitive mild TBI. Using transcriptomic and histological analyses, they reported decreased expression of genes involved in synaptic plasticity, neuronal communication, and neurotransmitter regulation alongside increased markers of cellular senescence in the cortex and hippocampus at 1 week post-injury. These molecular changes corresponded with impairments in spatial learning and memory as well as heightened anxiety-like behaviors, reinforcing the connection between synaptic alterations and functional deficits after repetitive injury (Aungst et al., 2014; Schwab et al., 2022).

Building on these findings, studies employing high-frequency repetitive head impact models have revealed further synaptic and network dysfunction, even in the absence of overt neuronal loss. Chapman et al. (2022) used a model of 30 repetitive head impacts in mice and found impaired early LTP in hippocampal CA1 neurons just 24 hours post-injury, along with disrupted neuronal ensemble dynamics and increased calcium transient frequency, indicating network-level dysfunction without neuronal loss. Complementing these results, Sloley et al. (2021) reported sustained dysregulation of synaptic genes, decreased LTP, reduced AMPA/NMDA receptor ratio, and diminished neuronal excitability in the hippocampus up to 1 month post-injury, with persistent cognitive impairments despite no synapse or neuron loss.

Beyond excitatory–inhibitory imbalance and synaptic dysfunction, rmTBI is also associated with progressive changes in synaptic density and neuronal survival, which differ across brain regions and may unfold gradually over time. Juan et al. (2022) reported that repetitive mild TBI (5 impacts) in mice led to significant neuronal loss in both the cortex and hippocampus, evidenced by reduced NeuN^+^ cell counts. Interestingly, despite this neuronal loss, synaptic markers such as PSD-95 remained unchanged, suggesting early neuronal stress without immediate synaptic degeneration. However, it is important to note that neuronal loss is not commonly observed in many other studies of repetitive mild TBI, which often report preserved neuronal numbers despite synaptic alterations (see **Additional Table 2** for a summary). Ogino et al. (2022) further supported this, showing persistent axonal injury with neuronal atrophy and hypertrophy but no evidence of cell death up to 28 days post-injury, highlighting neuronal survival despite ongoing damage. In a separate study, Boese et al. (2024), using a rat model of three repetitive mild TBIs, found that while white matter axonal degeneration was evident in the optic tract and lateral geniculate nucleus, synaptic density and postsynaptic marker levels (PSD-95) in these subcortical regions were largely preserved at 4 days post-injury. This suggests that early synaptic loss may not be a universal or immediate consequence of axonal injury in certain subcortical regions. Extending this temporal perspective, Markicevic et al. (2024) used SV2A PET and blood-oxygenation-level-dependent-fMRI imaging in mice subjected to repetitive mild TBI combined with chronic variable stress. They observed widespread reductions in synaptic density across cortical, thalamic, and hippocampal regions 6 weeks post-injury, despite no overt neuronal loss or structural damage on MRI. Concurrent blood-oxygenation-level-dependent-fMRI showed increased regional homogeneity, indicating compensatory hyperactivity within affected networks. These findings highlight that synaptic deficits following repetitive mild TBI may develop progressively over time, vary by brain region, and involve functional adaptations.

Expanding on previous observations from single mTBI, Chahin et al. (2025) provided a detailed analysis of microglial synaptic pruning following repetitive concussions, revealing prolonged and selective microglia-mediated engulfment of excitatory synapses with repeated injury and associated functional consequences. Specifically, unlike the transient increase in microglial engulfment seen after a single injury, as discussed earlier, repetitive mild TBIs induced sustained microglial engulfment of excitatory synapses (vGlut⁺) lasting from 48 hours up to 6 weeks post-injury in the sensorimotor cortex and hippocampus. Importantly, inhibitory synapses (vGat⁺) remained unaffected, consistent with the selectivity for excitatory inputs seen after a single injury, indicating that this selective pruning persists irrespective of injury frequency. Building on the earlier observation that single mTBI did not significantly alter spine type proportions, rmTBIs induced a transient increase in immature stubby spines at 1 week post-injury. This morphological change, not seen after a single injury, suggests temporary synaptic destabilization or delayed maturation during the prolonged microglial pruning phase. Importantly, total dendritic spine density in cortical layer II/III neurons remained stable, indicating preservation of overall synapse numbers despite these structural alterations (**Figure 4**). Other studies, such as Ratliff et al. (2020) reported reductions in dendritic length and spine density in the hippocampal dentate gyrus eight weeks following repetitive blast injury, demonstrating that longer-term dendritic structural deficits may develop in certain hippocampal subregions. Prolonged microglial pruning and spine morphological changes reported by Chahin et al. (2025) coincided with functional consequences, including impairments in spatial working memory and behavioral adaptation observed one week after injury. Importantly, no neuronal loss was detected even after repetitive injuries, emphasizing that these cognitive and behavioral impairments arise primarily from synaptic remodeling rather than overt cell death. Expanding on this, Eyolfson et al. (2022) demonstrated that repetitive injury during adolescence causes region-specific disruptions in synaptic pruning: excessive pruning and reduced spine density in the agranular insular cortex (prefrontal cortex), but impaired pruning with increased spine density in the motor cortex, particularly in adolescent males. These synaptic alterations were associated with motor coordination and recognition memory deficits, highlighting the role of disrupted pruning and microglial activity in age- and sex-dependent behavioral impairments. These preclinical data are mirrored by clinical trial data demonstrating that women had more severe and persistent cognitive and somatic symptoms than men at 12 months post-injury (TRACK-TBI study, including 2000 mTBI patients; Levin et al., 2021). These results led researchers to conclude that female sex is a risk factor for persistent cognitive and somatic post-concussion symptoms. Although women tend to have worse and longer-lasting symptoms, moderate female TBI patients have been reported to have a lower mortality rate than male patients of the same age (Ley et al., 2013), suggesting a protective effect of female sex hormones. However, clinical studies with both progesterone and testosterone did not confirm a protective effect of these hormones in the treatment of TBI (Wright et al., 2014; Ripley et al., 2020). Importantly, Levin et al. (2021) found that women aged 35-49 years had worse somatic symptoms than women aged 17-34 years. Macheda et al. (2024) reported similar findings in mice and showed that after mild TBI, older females had the poorest cognitive performance compared to their younger counterparts, older and younger males, as well as the greatest increase in CD45^+^ leukocytes in the corpus callosum, indicating progressive inflammatory engagement with chronic white matter degeneration. Microglia also remained elevated with age in both female and male mice, consistent with microglial priming (Perry and Holmes, 2014; Wangler and Godbout, 2023).

Overall, these studies collectively demonstrate that mild TBI induces significant synaptic and network dysfunction primarily through disrupted excitation–inhibition balance and selective microglial pruning of excitatory synapses. However, current research is limited by a focus on excitatory circuits, with a less detailed understanding of inhibitory interneuron diversity and their role in post-injury network stability. Also, while changes in long-term potentiation have been studied, other forms of synaptic plasticity, such as long-term depression and homeostatic adjustments, have not been explored as much. Moreover, long-term dynamics of synaptic recovery or decline, as well as sex- and age-dependent responses, remain insufficiently explored. Future studies would benefit from longitudinal approaches integrating molecular, cellular, and behavioral analyses to elucidate the mechanisms governing synaptic remodeling and microglia-related synaptic pruning. Such research could help develop targeted treatments that restore synaptic balance and reduce the risk of long-term cognitive problems after both single and repeated mild TBIs.

## Use of Biomarkers for the Diagnosis of Mild/Repetitive Traumatic Brain Injury

TBI biomarkers can be classified into two primary categories based on their temporal profiles: (1) markers exhibiting acute changes with rapid half-lives, such as S100B, GFAP, T-tau, and ubiquitin C-terminal hydrolase-L1 (UCH-L1); and (2) subacute markers that peak 7–12 days post-injury followed by gradual normalization, as observed with neurofilament light (NF-L) (Hossain et al., 2024). Because only ~10% of mTBI cases exhibit brain lesions detectable by computed tomography (CT), the development of reliable triage biomarkers could assist clinicians in identifying mTBI patients at risk of developing intracranial lesions who may require CT imaging (Lagerstedt et al., 2018, **Figure 3**).

### Acute biomarkers

S100B, a calcium-binding protein predominantly expressed in astrocytes, represents the most extensively studied biomarker for TBI assessment (Hossain et al., 2024; Thelin et al., 2017). Blood concentrations of S100B increase within 1 hour post-TBI, peaking within six hours of injury (Rodríguez-Rodríguez et al., 2012). The protein shows a short half-life in circulation, ranging from 30 minutes (Blomquist et al., 1997) to 97 minutes in the case of mTBI (Townend et al., 2006), with circulating levels influenced by age (Calcagnile et al., 2013). Therefore, it can only be used if the samples are collected within approximately 2 hours of the mTBI. While it is the most documented blood biomarker, its use remains limited by its low brain specificity (Thelin et al., 2017) and its short half-life. Nevertheless, the incorporation of S100B into clinical guidelines has demonstrated both cost-effectiveness and safety in reducing unnecessary CT scans (Undén et al., 2015; Calcagnile et al., 2016; Minkkinen et al., 2019). GFAP, a cytoskeletal protein primarily expressed in astrocytes (Eng et al., 1971), is released following injury-induced disruption of the astroglial cytoskeleton (Freire et al., 2023). GFAP has demonstrated prognostic utility in mTBI (Hossain et al., 2019), with day-of-injury plasma concentrations showing strong predictive value for unfavorable recovery (Korley et al., 2022; Hossain et al., 2024). Detectable in blood within 1 hour post-trauma (Welch et al., 2017), GFAP has an estimated half-life of 24 to 48 hours depending on injury severity and assay methodology (Thelin et al., 2019). Finally, UCH-L1, an abundant neuronal protease involved in ubiquitin metabolism (Liu et al., 2002), becomes detectable in blood within one hour of mTBI, peaks at approximately 8 hours post-injury, and has a half-life of 7 to 9 hours (Papa et al., 2016). However, some studies report conflicting findings regarding the ability of UCH-L1 to discriminate between healthy controls and mTBI patients (Posti et al., 2017; Dadas et al., 2018), with these discrepancies potentially attributable to methodological differences across studies (Hossain et al., 2024). Often used in combination with GFAP, UCH-L1 improves the diagnostic accuracy for mTBI (Reyes et al., 2023).

### Subacute biomarkers

NF-L is one of the five primary isoforms of neurofilaments, a type of intermediate filament (Samadzadeh and Sleator, 2025). Neurofilaments are abundant in large-caliber axons (Khalil et al., 2024). Importantly, NF-L is measurable in both blood and cerebrospinal fluid samples (Shahim et al., 2017). Shahim et al. (2020) reported higher NF-L levels in mTBI and concussion patients, and its increase has also been observed in contact sports athletes experiencing post-concussion symptoms (Shahim et al., 2017). NF-L typically peaks between 10 days and 6 weeks post-injury, with subacute levels showing particularly strong prognostic value (Shahim et al., 2020). Recently, the CENTER-TBI project, a large European initiative to improve patient care, indicated that day-of-injury NF-L measurements provide significant prognostic value for predicting incomplete recovery after mTBI (Helmrich et al., 2022). Interleukins are also important in the subacute phase following mTBI and rmTBI. IL-6, produced by glial cells and monocytes (Gadient and Otten, 1997), rises within 24 hours post-mTBI and correlates with CT-visible lesions (Chiollaz et al., 2024). Chiollaz et al. (2024), in a pediatric study, also reported 100% diagnostic accuracy for mTBI detection, with similar diagnostic value shown in adults (Thompson et al., 2020). IL-10, an anti-inflammatory cytokine (Iyer and Cheng, 2012), outperformed S100B in CT triage (27% specificity at 100% sensitivity) and shows acute post-mTBI elevation (Lagerstedt et al., 2018; Gill et al., 2018). One important limitation of interleukins as biomarkers is their lack of specificity for TBI.

### Emerging and specialized biomarkers

Tau, a microtubule-stabilizing neuronal protein (Alyenbaawi et al., 2020), shows abnormal accumulation following rmTBI that may contribute to chronic traumatic encephalopathy (Puangmalai et al., 2025). A large imaging study of pediatric mTBI patients found that blood-based tau levels correlated with injury severity and intracranial lesion burden (Mayer et al., 2025). Phosphorylated tau (p-tau181) shows acute elevation in mTBI (peaking within 18 to 48 hours), with moderate accuracy for patients with positive neuroimaging findings (Devoto et al., 2023). However, tau pathology is polymorph-specific, meaning that not all tau aggregates behave homogeneously, therefore restricting clinical applications.

Despite promising biomarker performance, routine clinical use remains challenging due to pre-analytical handling (e.g., hemolysis, processing delays, and storage/freeze–thaw cycles) and analytical variability (antibody variability, calibration standards), among other factors, all contributing to poor reproducibility. Insufficient validation across age and polytrauma populations also hampers the establishment of universally agreed reference values. In addition, narrow temporal windows and the need for serial sampling can limit rapid risk stratification. The efficient implementation of the biomarkers presented in this review would therefore require method standardization and further research in pediatric, older adult, and polytrauma populations.

## Conclusions

Management of mild TBI and repetitive mild TBI is primarily supportive, with an emphasis on symptom monitoring, gradual return to activity, and rehabilitation. There is currently no approved pharmacotherapy for mild/repetitive mild TBI, and existing treatments focus solely on symptom management for headaches, cognitive, mood, or sleep symptoms. However, preclinical new studies are emerging that use models of mild/repetitive mild TBI. For example, research conducted by Kim et al. (2022) investigated the potential therapeutic role of human embryonic stem cell-derived cerebral organoids in treating mTBI. In their study, these organoids were transplanted onto the injured cortices of mice 1 week post-injury. This intervention led to reduced neuronal cell death and stimulated neurogenesis within the hippocampal dentate gyrus and subventricular zone. The grafts also promoted angiogenesis and predominantly differentiated into TUJ1-positive immature neurons. Behavioral improvements, such as enhanced performance in the novel object recognition test, suggested a restoration of cognitive function. In a related investigation, Kim et al. (2023) explored a hydrogel-based delivery system for mouse neural stem cell spheroids. Transplanted into the damaged cortices of mTBI-injured mice, effective cell engraftment, survival, and differentiation into neurons were observed, as well as improved cognitive function. Further, Wang et al. (2024) examined the use of bone marrow-derived mesenchymal stem cells in a rat model of rmTBI. Histological analyses conducted months after injury revealed that untreated animals exhibited white matter thinning, cognitive and sensorimotor deficits, and increased microglial TNF-α expression that were improved by the treatment. Additional treatments include the use of mitoquinone, a mitochondria-targeted antioxidant (Murphy and Smith, 2007; Smith et al., 2003), or memantine, an NMDA receptor antagonist. Mitoquinone, administered shortly after injury and biweekly for 1 month, improved motor and cognitive performance, reduced microgliosis and astrocytosis, and mitigated axonal and dendritic damage in the hippocampus and cortex (Tabet et al., 2022). MacLean et al. (2023) investigated the effects of memantine on cortical spreading depolarizations, which are increasingly recognized as contributors to TBI pathology. The study suggested that memantine may exert neuroprotective effects in the context of mTBI by attenuating cortical spreading depolarization activity and associated excitotoxic mechanisms. Finally, a recent study using psilocybin, known to reduce neuroinflammation and enhance neuroplasticity, showed, in a model of mTBI in female rats, restoration of normal vascular reactivity and functional connectivity, reduction of phosphorylated tau buildup, increased levels of brain-derived neurotrophic factor and its receptor TrkB, and modulation of lipid signaling molecules (Brengel et al., 2025). All these preclinical studies highlight the current intense focus on experimental mTBI and rmTBI, whereas for patients, the standard of care remains rest, symptom management, and rehabilitation or supportive care. In addition, this review also highlights microglia as additional therapeutic targets for mild and repetitive mild TBI. Understanding the temporal dynamics of microglial activation, functional and structural changes, receptor signaling, metabolic reprogramming, and microglia-mediated synapse removal is critical for identifying precise therapeutic windows. Explicitly linking these mechanisms to targeted interventions offers promising opportunities to modulate microglial phenotypes, promote synaptic recovery, and attenuate pathological pruning to ultimately improve cognitive outcomes. Future research should therefore focus on deciphering the timing and pathways of microglial priming and transitional states to optimize treatment timing and develop therapies that balance neuroprotection and immune regulation in m- and rmTBI (Huang et al., 2021; Scott et al., 2021).

## Additional files:

***Additional Table 1:*** *Summary of papers investigating structural and functional microglial changes following mTBI and rmTBI between 2014 and 2025.*

***Additional Table 2:*** *Summary of papers investigating neuronal and synaptic changes following mTBI and rmTBI between 2014 and 2025.*

## Data Availability

*Not applicable.*
